# Identification of baseline gene expression signatures predicting therapeutic responses to three biologic agents in rheumatoid arthritis: a retrospective observational study

**DOI:** 10.1186/s13075-016-1052-8

**Published:** 2016-07-19

**Authors:** Seiji Nakamura, Katsuya Suzuki, Hiroshi Iijima, Yuko Hata, Chun Ren Lim, Yohei Ishizawa, Hideto Kameda, Koichi Amano, Kenichi Matsubara, Ryo Matoba, Tsutomu Takeuchi

**Affiliations:** DNA Chip Research Inc., 1-15-1 Kaigan, Suzuebaydium 5F, Minato-ku, Tokyo 105-0022 Japan; Division of Rheumatology, Department of Internal Medicine, Keio University School of Medicine, 35 Shinanomachi, Shinjuku-ku, Tokyo 160-8582 Japan; Division of Rheumatology, Department of Internal Medicine, Toho University Ohashi Medical Center, 2-17-6 Ohashi, Muguro-ku, Tokyo 153-8515 Japan; Department of Rheumatology and Clinical Immunology, Saitama Medical Center, Saitama Medical University, 1981 Tsujido-machi Kamoda, Kawagoe-shi, Saitama 350-8550 Japan

**Keywords:** Rheumatoid arthritis, bDMARDs (biologic agents), Prediction, Gene expression

## Abstract

**Background:**

According to EULAR recommendations, biologic DMARDs (bDMARDs) such as tumor necrosis factor inhibitor, tocilizumab (TCZ), and abatacept (ABT) are in parallel when prescribing to rheumatoid arthritis (RA) patients who have shown insufficient response to conventional synthetic DMARDs. However, most prediction studies of therapeutic response to bDMARDs using gene expression profiles were focused on a single bDMARD, and consideration of the results from the perspective of RA pathophysiology was insufficient. The aim of this study was to identify the specific molecular biological features predicting the therapeutic outcomes of three bDMARDs (infliximab [IFX], TCZ, and ABT) by studying blood gene expression signatures of patients before biologic treatment in a unified test platform.

**Methods:**

RA patients who responded inadequately to methotrexate and were later commenced on any one of IFX (*n* = 140), TCZ (*n* = 38), or ABT (*n* = 31) as their first biologic between May 2007 and November 2011 were enrolled. Whole-blood gene expression data were obtained before biologic administration. Patients were categorized into remission (REM) and nonremission (NON-REM) groups according to CDAI at 6 months of biologic therapy. We employed Gene Set Enrichment Analysis (GSEA) to identify functional gene sets differentially expressed between these two groups for each biologic. Then, we compiled “signature scores” for these gene sets, and the prediction performances were assessed.

**Results:**

GSEA showed that inflammasome genes were significantly upregulated with IFX in the NON-REM group compared with the REM group. With TCZ in the REM group, B-cell-specifically expressed genes were upregulated. RNA elongation, apoptosis-related, and NK-cell-specifically expressed genes were upregulated with ABT in the NON-REM group. Logistic regression analyses showed that “signature scores” of inflammasomes, B-cell-specifically expressed, and NK-cell-specifically expressed genes were significant, independently predictive factors for treatment outcome with IFX, TCZ, and ABT, respectively. The AUCs of ROC curves of these signature scores were 0.637, 0.796, and 0.768 for IFX, TCZ, and ABT, respectively.

**Conclusions:**

We have identified original gene expression predictive signatures uniquely underlying the therapeutic effects of IFX, TCZ, and ABT. This is, to our knowledge, the first attempt to predict therapeutic effects of three drugs concomitantly using a unified gene expression test platform.

**Electronic supplementary material:**

The online version of this article (doi:10.1186/s13075-016-1052-8) contains supplementary material, which is available to authorized users.

## Background

Methotrexate (MTX) and biologic disease-modifying antirheumatic drugs (bDMARDs) have brought therapeutic success to most but not all patients with rheumatoid arthritis (RA). Optimization of treatment for individual patients and development of novel therapies are eagerly anticipated. A good effort regarding the former is to establish a standard methodology to determine which bDMARDs to prescribe. As the molecular target of each bDMARD is distinct, each effective treatment should link to changes in one or several particular biological processes that are ultimately manifested in a disease state. Despite this understanding and supporting evidence, current concepts of prescription of bDMARDs are unsatisfactory. According to the European League Against Rheumatism (EULAR) recommendations, tumor necrosis factor (TNF) inhibitor, tocilizumab (TCZ), and abatacept (ABT) are in parallel when determining first biologics to use for patients with RA who have shown inadequate response to conventional synthetic DMARDs (csDMARDs) [1]. Development of a methodology to determine effective therapy using bDMARDs is definitely essential.

Most prediction studies of therapeutic response to bDMARDs using gene expression profiles of blood samples have been focused on a single biologic [2–9], and to date no report of multiple drugs studied in parallel is available. Furthermore, study designs have varied, thus rendering translational studies very challenging.

Difficulty in reproducing gene expression studies has also plagued this area of study, partly due to nonuniformities of study design but also to data processing itself [10]. Instead of incorporating existing biological knowledge, analysis rarely extends beyond the individual gene level to explain how the biomarker findings are associated with modes of action related to targeted therapies for RA. Furthermore, to establish a robust model using gene expression, it is essential to interpret the results as effects of a collective network of related genes rather than of the gene per se. In this context, Gene Set Enrichment Analysis (GSEA) [11], which was shown to detect differentially expressed functional gene sets, should be a promising approach.

In this study, to identify therapeutic efficacy markers of three bDMARDs (infliximab [IFX], TCZ, and ABT) targeted at different molecules, we took the aforementioned problems into consideration. We designed a unified test platform in which the subject recruitment criteria, treatment response evaluation, and assay system platform are well defined. GSEA is employed to identify and annotate the gene signatures associated with each biologic. The prediction performance, biological interpretation, and utility of each gene signature are presented.

## Methods

### Patients and evaluation of effectiveness

The diagnosis of RA in the present study was based on the 1987 revised criteria of the American College of Rheumatology (ACR) for the classification of RA or on the 2010 ACR/EULAR classification criteria. We enrolled patients with RA who responded inadequately to MTX (≥6 mg/week) and were commenced on any one of IFX, TCZ (2008), or ABT (2010) as their first biologic between May 2007 and November 2011 at Keio University Hospital and Saitama Medical University Saitama Medical Center. Biologics were administered according to the guidelines set by the Japan College of Rheumatology (http://www.ryumachi-jp.com/guideline.html [in Japanese]). Therapeutic outcomes were defined as achieving remission (REM; defined as clinical disease activity index [CDAI] ≤2.8) or not achieving remission (NON-REM) on the basis of CDAI at 6 months of biologic therapy, since other disease activity indexes such as the disease activity score in 28 joints (DAS28) incorporate inflammatory factors such as C-reactive protein or erythrocyte sedimentation rate (ESR), which may overestimate the efficacy of TCZ [12, 13]. Patients who discontinued biologic therapy by 6 months due to insufficient effects (*n* = 5) or adverse events (*n* = 1) were classified as NON-REM (Additional file 1). The CDAI of all six cases were >2.8 as determined using the last observation carried forward (LOCF) method. Written informed consent was obtained from all patients in accordance with the Declaration of Helsinki protocol, and the study protocol was approved by the institutional review boards at Keio University and Saitama Medical University.

### RNA extraction

Before administration of a biologic agent, blood samples were collected in PAXgene Blood RNA tubes [14] (PreAnalytiX, Hombrechtikon, Switzerland). Total RNAs were extracted using PAXgene Blood RNA kits (PreAnalytiX) following the manufacturer’s instructions. Total RNA quantity and quality were determined using a NanoDrop 1000 spectrophotometer (NanoDrop Products/Thermo Fisher Scientific, Wilmington, DE, USA) and an Agilent 2100 Bioanalyzer (Agilent Technologies, Santa Clara, CA, USA). All RNA samples fulfilled both of the following criteria: RNA integrity >6.5 and optical density at 260/280 nm >1.6.

### Gene expression measurements

Cyanine 3-labeled complementary RNAs (cRNAs) were synthesized using QuickAmp Labeling Kits (Agilent Technologies). The cRNAs were hybridized at 65 °C for 17 h to Whole Human Genome 44 K Microarrays (design ID 014850; Agilent Technologies). After being washed, the microarrays were scanned using an Agilent DNA microarray scanner (Agilent Technologies). Intensity values of each scanned feature were quantified using Agilent Feature Extraction software (Agilent Technologies). The raw microarray data are deposited in the National Center for Biotechnology Information Gene Expression Omnibus database under accession number [GSE78068]. We applied rank-based quantile normalization to the raw signal data using R software version 3.0.2. Next, probes were filtered based on preexisting annotation with gene symbol and signal intensity (called “present” in more than 50 samples according to GeneSpring software [Agilent Technologies]). For genes with more than one probe, we adopted the probe that had the highest signal intensity. The final number of probes used for subsequent analysis was 14,718.

### GSEA

We employed GSEA to study the molecular biological features of the REM and NON-REM groups associated with each biologic therapy. GSEA is a computational method that determines whether a set of genes defined a priori shows statistically significant, concordant differences between two biological states [11].

We used GSEA v2.1.0, and input data comprised three sets of data matrices (i.e., 14,718 genes × 140 samples [IFX], 14,718 genes × 38 samples [TCZ], and 14,718 genes × 31 samples [ABT]). Two lists of gene sets were used as sets of genes defined a priori (i.e., Reactome gene sets in the Molecular Signatures Database [11]), containing 674 pathways and integrated lists of blood cell type-specific expressed gene sets published by Watkins et al. [15] and Allantaz et al. [16]. The integrated lists have 16 blood cell type-specific expressed gene sets (Additional file 2). Permutation type was set as “phenotype,” and the number of permutations was 1000. The population gene set for analysis was 14,718, and the metrics for ranking genes were the signal-to-noise ratios. Gene set size filters were in default settings, where the minimum was 15 and the maximum was 500. Gene sets with a nominal *p* value <0.05 and a false discovery rate <0.1 were considered significant. Then, we defined “core genes” as the subset of genes that contributed most to the GSEA enrichment score.

### Real-time quantitative reverse transcription polymerase chain reaction

Real-time quantitative reverse transcription-polymerase chain reactions (qRT-PCRs) were performed for 11–13 samples of each biologic where total RNAs were adequate. Genes measured were *APP*, *AIM2*, *NLRC4*, *MEFV*, and *BCL2L1* for inflammasomes (signature of IFX); *PLEKHG1*, *AFF3*, *FCER2*, *UGT8*, and *CD22* for specific CD19 (signature of TCZ); and *BNC2*, *CD160*, *PDGFRB*, *LIM2*, and *KIR3DL2* for specific CD56 (signature of ABT). We designed custom RT^2^ Profiler PCR Arrays (QIAGEN, Valencia, CA, USA), and the assay was performed according to the manufacturer’s instructions. Essentially, 500 ng of total RNA of each sample was used to synthesize complementary DNA using the RT^2^ HT First Strand Kit (QIAGEN). qRT-PCR was performed using the Applied Biosystems 7500 Fast Dx Real-Time PCR System (Thermo Fisher Scientific, Foster City, CA, USA). The relative expression of each gene was quantified by measuring cycle threshold (Ct) values and normalizing against *GUSB*.

### Calculation of signature score

The scoring system we used, which is clinically applicable to each patient, is shown in Additional file 3. Briefly, each core gene that belonged to a target gene set was standardized using a *z*-score transformation based on all 209 patients’ data, and then the average of *z*-scores of all core genes was defined as the “signature score” of the gene set for each patient.

### ROC analysis

ROC (receiver operating characteristic) analysis was conducted using the signature score compared with REM versus NON-REM category, and AUC of the ROC curve was determined. We applied the same sample group that was used to construct the gene signature. NON-REM was defined as “positive.” The sensitivity, specificity, positive predictive value (PPV), and negative predictive value were determined at the optimal cutoff value (threshold) from the ROC curve. Analysis was performed using R software version 3.0.2.

### Statistical analysis

The CDAI of six samples where administration was terminated before 6 months (see "Patients and evaluation of effectiveness" section) was estimated using the LOCF method. The Kruskal-Wallis test, Wilcoxon’s rank-sum test, or Student’s *t* test was performed for numerical variables. For categorical variables, Fisher’s exact test was conducted. The associations between CDAI remission at 6 months of biologic therapy and signature scores were evaluated using univariate and multivariate logistic regression analyses (Firth’s penalized likelihood method [17]). For multivariate analyses, we adjusted for marginally significant (*p* < 0.1) univariate factors as shown in Additional file 4. However, owing to the strong correlation with CDAI (in IFX and TCZ analysis) or concomitant steroid use (in ABT analysis), we did not adjust 28 tender joint count (TJC28), 28 swollen joint count (SJC28), patient global assessment (PtGA), physician global assessment (PhGA), DAS28-ESR, and Simplified Disease Activity Index (SDAI) in IFX analysis; SJC28, DAS28-ESR, and SDAI in TCZ analysis; or concomitant steroid dose in ABT analysis.

In this study, *p* < 0.05 was considered significant. *p* Values derived from these analyses were not adjusted for multiple testing. All statistical analyses were performed with R software version 3.0.2.

## Results

### Baseline clinical characteristics and therapeutic response to each biologic therapy

There were 140, 38, and 31 cases (total 209) of IFX, TCZ, and ABT, respectively. The baseline characteristics of the enrolled patients of three biologic groups are shown in Table 1. The median age of the patients from whom the 209 samples were derived was 59 years, and their disease duration was 3.3 years. Coadministration of MTX had a median volume of 8 mg, and the median CDAI was 21.7. Among the biologic agents, the subjects given ABT were older, and coadministration of MTX was slightly higher in the IFX group.

**Table 1:** Patients’ baseline demographics and characteristics

	All	IFX	TCZ	ABT	*p* Value^a^
Number of patients	209	140	38	31	–
Female sex, *n* (%)	172 (82.3 %)	113 (80.7 %)	34 (89.5 %)	25 (80.6 %)	0.46626
Age, yr, median (IQR)	59.0 (47.0–66.0)	57.5 (46.0–64.3)	56.0 (44.8–64.0)	67.0 (62.0–74.0)	0.00003
Disease duration, yr, median (IQR)	3.3 (1.1–10.5)	3.3 (1.1–10.3)	4.2 (1.5–9.4)	2.4 (0.5–14.5)	0.86013
Concomitant drug use					
Steroid use, *n* (%)	80 (38.3 %)	56 (40.0 %)	14 (36.8 %)	10 (32.3 %)	0.75247
Steroid dose, mg/day, median (IQR)	0 (0–5.0)	0 (0–5.0)	0 (0–3.0)	0 (0–2.5)	0.34861
MTX dose, mg/week, median (IQR)	8.0 (8.0–8.0)	8.0 (8.0–10.0)	8.0 (6.0–8.0)	8.0 (6.0–8.0)	0.00271
csDMARD use^b^ (except MTX), *n* (%)	29 (13.9 %)	20 (14.3 %)	5 (13.2 %)	4 (12.9 %)	1.00000
Serological markers					
RF positivity, *n* (%)	154 (74.8 %)^c^	103 (74.1 %)^d^	28 (73.7 %)	23 (79.3 %)^e^	0.88544
RF titer, median (IQR)	55 (15–115)^c^	53 (14–115)^d^	54 (17–115)	77 (22–106)^e^	0.94307
ACPA positivity, *n* (%)	85 (85.9 %)^f^	51 (85.0 %)^g^	13 (100 %)^h^	21 (80.8 %)^i^	0.31205
ACPA titer, median (IQR)	85.6 (12.8–100)^f^	100 (13.3–100)^g^	83 (42.9–100)^h^	62.1 (9.8–100)^i^	0.89773
TJC28, median (IQR)	6.0 (2.0–8.0)	6.0 (2.0–8.3)	6.0 (3.0–8.8)	6.0 (3.0–8.0)	0.68798
SJC28, median (IQR)	6.0 (3.0–11.0)	7.0 (3.8–11.0)	6.0 (4.0–8.0)	5.0 (3.0–8.0)	0.46376
PtGA, mm, median (IQR)	53 (28–72)	52 (27–72)	52 (31–67)	63 (41–73)	0.34811
PhGA, mm, median (IQR)	43 (30–60)	43 (29–63)	43 (33–60)	45 (31–56)	0.91700
CRP, mg/dl, median (IQR)	0.9 (0.4–2.4)	1.0 (0.4–2.6)	0.6 (0.2–2.0)	0.8 (0.4–2.0)	0.19092
ESR, mm/h, median (IQR)	42 (25–69)	45 (28–69)	37 (22–60)	37 (23–74)	0.27517
DAS28-ESR, median (IQR)	5.3 (4.4–6.1)	5.3 (4.4–6.2)	5.1 (4.5–5.8)	5.5 (4.7–6.1)	0.65899
SDAI score, median (IQR)	22.5 (16.2–31.3)	23.2 (15.6–32.2)	22.1 (16.8–28.5)	23.3 (16.8–31.5)	0.96973
CDAI score, median (IQR)	21.7 (14.9–28.9)	21.9 (14.6–29.6)	20.6 (16.4–26.0)	22.8 (15.1–28.5)	0.99430

*Abbreviations:* *IFX* infliximab, *TCZ* tocilizumab, *ABT* abatacept, *IQR* interquartile range, *MTX* methotrexate, *csDMARD* conventional synthetic disease-modifying antirheumatic drug, *RF* rheumatoid factor, *ACPA* anticyclic citrullinated peptide antibodies, *TJC28* 28 tender joint count, *SJC28* 28 swollen joint count, *PtGA* patient global assessment, *PhGA* physician global assessment, *CRP* C-reactive protein, *ESR* erythrocyte sedimentation rate, *DAS28* disease activity score in 28 joints, *SDAI* simplified disease activity index, *CDAI* clinical disease activity index

^a^Kruskal-Wallis test was used for numerical variables to evaluate the differences between the three drug groups. For categorical variables, Fisher’s exact test was used. *p* < 0.05 was considered statistically significant

^b^Including salazosulfapyridine, bucillamine, tacrolimus, d-penicillamine, actarit, and azathioprine

^c^Available for 206 of 209

^d^Available for 139 of 140

^e^Available for 29 of 31

^f^Available for 99 of 209

^g^Available for 60 of 140

^h^Available for 13 of 38

^i^Available for 26 of 31

**Fig. 1:**
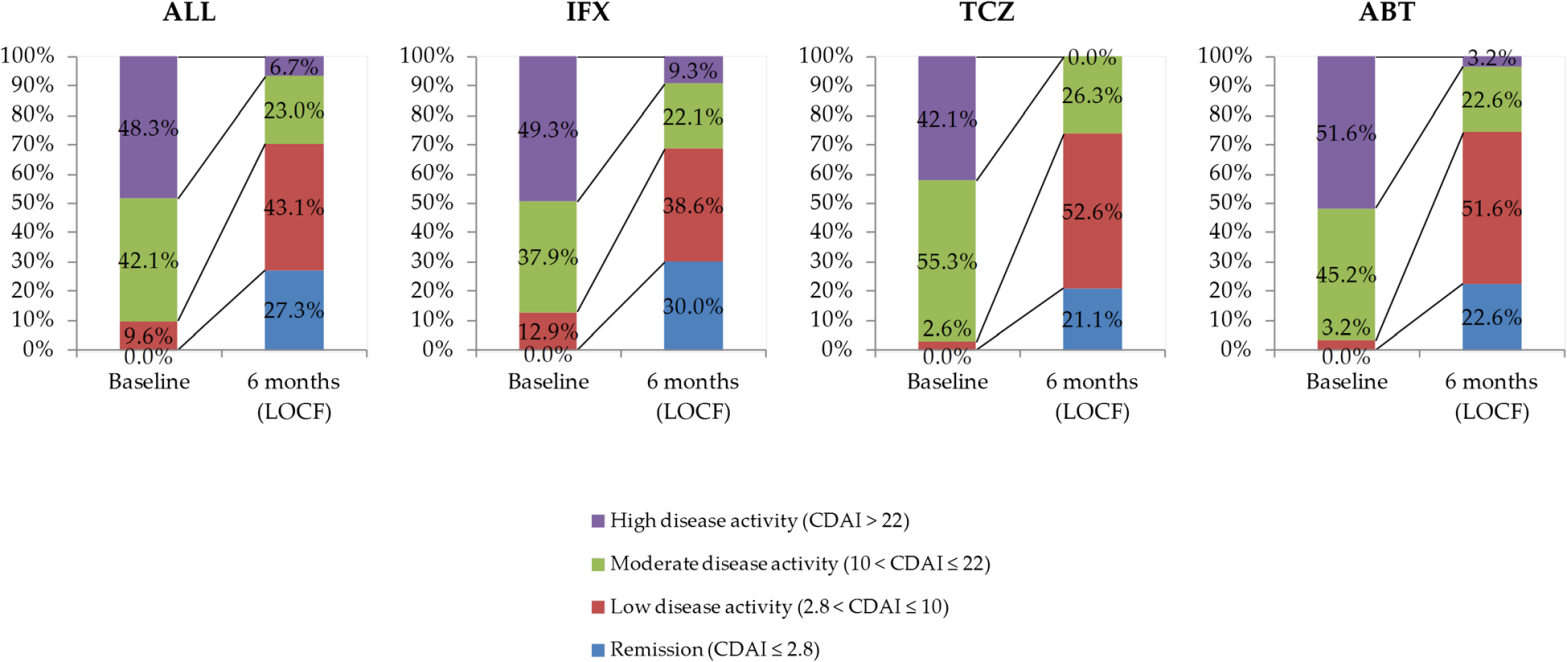
Disease activity based on CDAI at baseline and 6 months of biologic therapy. * CDAI* clinical disease activity index, *LOCF* last observation carried forward, *IFX* infliximab, *TCZ* tocilizumab, *ABT* abatacept

Administration was terminated by 6 months for IFX (*n* = 1), TCZ (*n* = 3), and ABT (*n* = 1) due to insufficient effect (Additional file 1). In addition, there was an adverse effect case in TCZ. These cases were subsequently classified as NON-REM. For all 209 cases, 27.3 % achieved remission at 6 months of biologic therapy (Fig. 1). The remission rates for IFX, TCZ, and ABT were 30.0 %, 21.1 %, and 22.6 %, respectively.

Differences in baseline characteristics between the REM and NON-REM groups are shown in Additional file 4. There were significant differences in female proportion (*p* = 0.034) and concomitant use of csDMARDs besides MTX (*p* = 0.016) between the REM and NON-REM groups for IFX. SJC28, PtGA, PhGA, DAS28-ESR, SDAI, and CDAI were also significantly higher for IFX in the NON-REM group. For TCZ, the NON-REM group had a higher DAS28-ESR (*p* = 0.045). The duration of disease for the ABT group was longer in the NON-REM group (*p* = 0.003).

### Baseline gene expression features underlying REM and NON-REM

We used GSEA to identify the molecular biological features of the REM and NON-REM groups for each biologic therapy. GSEA is a powerful analytical method to detect modest but coordinated changes in the expression of groups of functionally related genes. Table 2 summarizes the results of our GSEA (see also Additional files 5, 6, 7 and 8). In Reactome gene set analyses, “inflammasomes” in IFX, “elongation arrest and recovery,” “regulation of apoptosis,” and “formation of RNA polymerase II (RNA pol II) elongation complex” in ABT showed upregulated expression patterns for NON-REM. In blood cell gene set analysis, signatures related to B cells, such as “specific CD19” and “B cell-induced” showed upregulated expression patterns in the TCZ group for REM. In the ABT group, signatures related to natural killer (NK) cells, such as “specific CD56” and “NK cell-induced” showed upregulated expression patterns for NON-REM. The top-performing genes for each gene set were validated using qRT-PCR (Additional file 9).

**Table 2:** Gene Set Enrichment Analysis results

Gene set category	Drug	Direction of regulation	Gene set name	Size^a^	NES^b^	NOM *p* value^c^	FDR *q* value^d^
Reactome gene sets	IFX	REM > NON-REM	–	–	–	–	–
		NON-REM > REM	Inflammasomes (M1072)	16	1.95	<0.00001	0.07489
	TCZ	REM > NON-REM	–	–	–	–	–
		NON-REM > REM	–	–	–	–	–
	ABT	REM> NON-REM	–	–	–	–	–
		NON-REM > REM	Elongation arrest and recovery (M810)	27	1.91	0.00180	0.08880
			Regulation of apoptosis (M733)	54	1.96	0.00192	0.09083
			Formation of RNA pol II elongation complex (M805)	38	1.85	<0.00001	0.09138
Blood cell gene sets	IFX	REM > NON-REM	–	–	–	–	–
		NON-REM > REM	–	–	–	–	–
	TCZ	REM > NON-REM	Specific CD19 (Watkins et al., 2009 [15])	140	−1.70	0.00602	0.02646
			B-cell-induced (Allantaz et al., 2012 [16])	57	−1.56	0.01603	0.08882
		NON-REM > REM	–	–	–	–	–
	ABT	REM > NON-REM	–	–	–	–	–
		NON-REM > REM	Specific CD56 (Watkins et al., 2009 [15])	51	1.60	0.02390	0.02615
			NK-cell-induced (Allantaz et al., 2012 [16])	78	1.63	0.00403	0.02861

*Abbreviations:*  *IFX* infliximab, *TCZ* tocilizumab, *ABT* abatacept, *REM* patients with CDAI remission (defined as CDAI ≤2.8) at 6 months of biologic therapy, *NON-REM* patients without CDAI remission at 6 months of biologic therapy, *CDAI* clinical disease activity index, *NK* natural killer, *RNA pol II* RNA polymerase II

A nominal *p* value <0.05 and false discovery rate *q* value <0.1 were considered statistically significant

^a^Number of genes found in the gene set from the expression dataset

^b^Normalized enrichment score is the enrichment score for the gene set after it has been normalized across analyzed gene sets

^c^Nominal *p* value is the statistical significance of the enrichment score. Nominal *p* value is not adjusted for gene set size or multiple hypothesis testing

^d^False discovery rate is the estimated probability that the normalized enrichment score represents a false-positive finding

### Signature scores and therapeutic responses

**Fig. 2:**
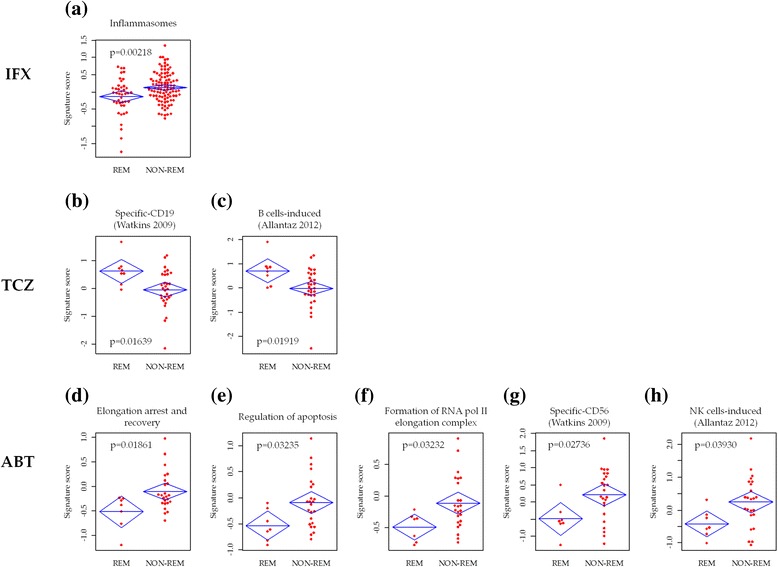
Comparisons of signature scores between REM and NON-REM for infliximab (IFX) (**a**), tocilizumab (TCZ) (**b**, **c**), and abatacept (ABT) (**d**–**h**). Distributions of the values, mean, and upper and lower limits of 95 % confidence intervals for the mean are shown. *p* Values were determined using Student’s *t* test. *p* < 0.05 was considered statistically significant. *REM* patients with CDAI remission (defined as CDAI ≤2.8) at 6 months of biologic therapy, *NON-REM* patients without CDAI remission at 6 months of biologic therapy, *CDAI* clinical disease activity index, *RNA pol II* RNA polymerase II, *NK* natural killer

We compiled “signature scores” based on gene sets identified using GSEA to evaluate individual gene expression profiles (see Methods section). Signature scores generated were able to significantly differentiate REM and NON-REM (Fig. 2a–h) and thus could serve as a system to predict each individual’s prospective therapeutic outcome.

We found overlapping genes especially within gene sets of the TCZ group and gene sets of the ABT group (Additional files 7 and 8). Correlation analysis of signature scores of these gene sets indeed confirmed redundancy (Additional file 10). For the predictive signature of TCZ, “specific CD19” and “B cell-induced” were consolidated as “specific CD19” (Pearson’s correlation coefficient 0.99). For ABT, “elongation arrest and recovery,” “regulation of apoptosis,” and “formation of RNA pol II elongation complex” were closely related (correlation coefficients 0.77, 0.91, and 0.84, respectively), while “specific CD56” and “NK cell-induced” also shared a high correlation (correlation coefficient 0.96). We thus subsequently focused on “elongation arrest and recovery” and “specific CD56.”

Logistic regression analyses of signature score as a univariate independent variable and CDAI remission as a dependent variable showed that each signature score was significant (Table 3). In multivariate analyses where the clinical background of REM and NON-REM (Table 1) was also taken into account, signature scores remained significant. We also found that “specific CD56” was more significant than “elongation arrest and recovery” in ABT, and thus we concentrated on “specific CD56” in subsequent analysis.

**Table 3:** Logistic regression analyses using signature scores to predict CDAI nonremission at 6 months of biologic therapy

Drug	Gene set	Univariate analysis			Multivariate analysis^a^		
		OR	95 % CI	*p* Value	OR	95 % CI	*p* Value
IFX							
	Inflammasomes	1.81	(1.23–2.78)	0.00382	1.72	(1.14–2.71)	0.00873
TCZ							
	Specific CD19 (Watkins et al., 2009 [15])	0.24	(0.05–0.72)	0.02677	0.16	(0.02–0.71)	0.01327
ABT							
	Elongation arrest and recovery	5.73	(1.47–49.11)	0.04488	6.85	(1.09–4426.71)	0.03309
	Specific CD56 (Watkins et al., 2009 [15])	3.25	(1.18–12.43)	0.04179	6.46	(1.60–88.39)	0.00388

*Abbreviations:* *CDAI* clinical disease activity index, *IFX* infliximab, *TCZ* tocilizumab, *ABT* abatacept, *OR* odds ratio, *CI* confidence interval, *csDMARD* conventional synthetic disease-modifying antirheumatic drug, *MTX* methotrexate, *ESR* erythrocyte sedimentation rate

^a^Multivariate analysis adjusted for significant (*p* < 0.1) variables as in Additional file 4 (IFX: female, concomitant csDMARD use [except MTX], ESR, CDAI; TCZ: disease duration, CDAI; ABT: disease duration, concomitant steroid use)

### Evaluation of prediction performance using signature score

**Fig. 3:**
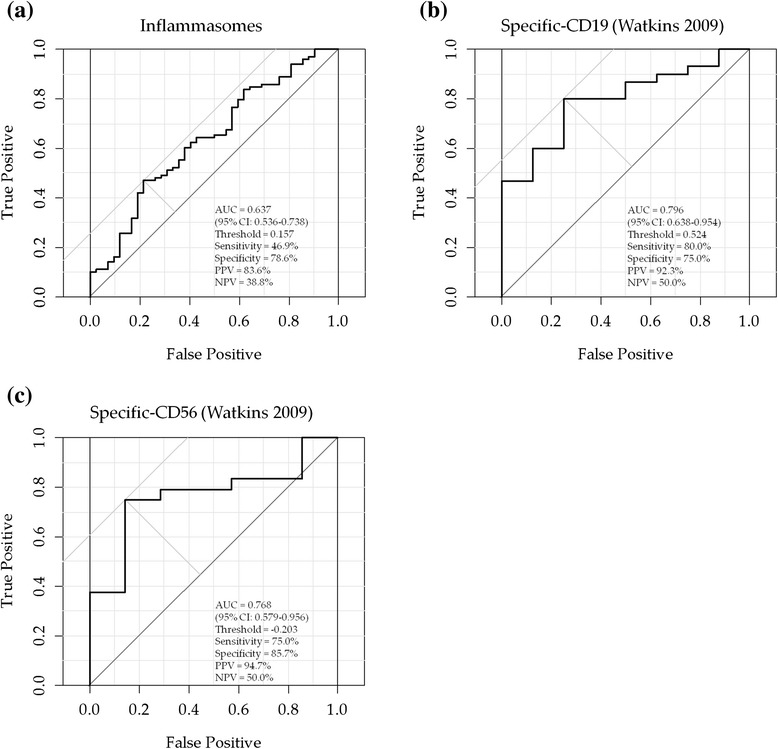
Receiver operating characteristic curve analyses for the prediction of CDAI nonremission at 6 months of biologic therapy. **a** infliximab. **b** tocilizumab. **c** abatacept. *CDAI* clinical disease activity index, *AUC* area under the curve, *CI* confidence interval, *PPV* positive predictive value, *NPV* negative predictive value. “Positive” means CDAI nonremission at 6 months of biologic therapy

ROC analysis was performed using signature scores (Fig. 3). The AUCs of signature scores to predict NON-REM based on CDAI were 0.637 (IFX, signature: inflammasomes), 0.796 (TCZ, signature: specific CD19) and 0.768 (ABT, signature: specific CD56). Notably, for all biologic agents, the PPV were high (IFX 83.6 %, TCZ 92.3 %, ABT 94.7 %).

### Overview of classification of all 209 samples using signature scores

**Fig. 4:**
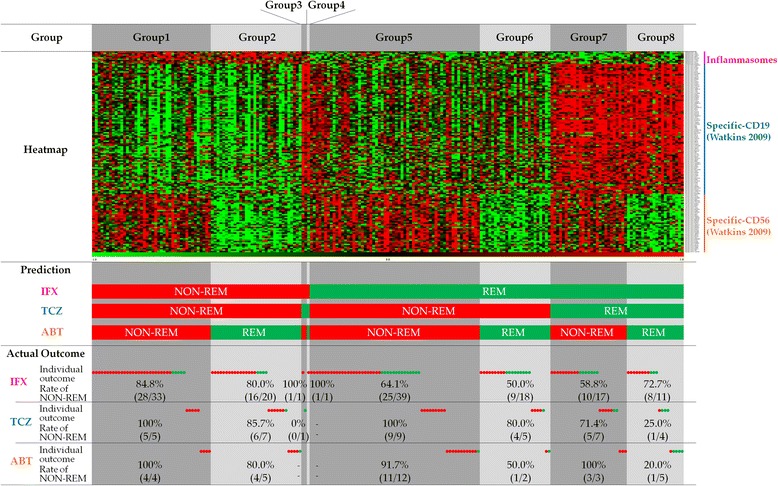
An overview of classification of all 209 samples using 3 signature scores. All 209 samples could be classified into 8 groups, based on the binary variables derived from the signature scores of the three gene sets (i.e., inflammasome-, specific CD19-, and specific CD56-related). The threshold for binary call was determined using ROC curve analysis. *Top panel:* Heat map of 209 samples based on expression patterns of the three core gene sets. The heat map was performed based on relative expression levels (*z*-scores) of core genes using TIGR MultiExperiment Viewer Software (http://www.tm4.org/). *Middle panel:* Prediction results using signature scores. Patient outcomes were predicted using signature score and grouped as remission (REM) and nonremission (NON-REM) as indicated in *green* and *red*, respectively. *Bottom panel:* Actual remission status based on CDAI at 6 months of biologic therapy. In “Individual outcome,” *green* and *red* circles represent actual individual cases achieving or not achieving remission, respectively. In “Rate of NON-REM,” the actual number of cases and the nonremission rates are indicated for all eight groups. *REM* patients with CDAI remission at 6 months of biologic therapy, *NON-REM* patients without CDAI remission at 6 months of biologic therapy, *CDAI* clinical disease activity index, *IFX* infliximab, *TCZ* tocilizumab, *ABT* abatacept

A heat map using core genes of inflammasomes, specific CD19, and specific CD56 revealed that all 209 samples analyzed in this study could be distributed into 8 groups, based on the signature score cutoff points determined in ROC curve analyses (Fig. 4). When these groups were compared with regard to actual therapeutic outcomes, the proportions of NON-REM in groups 1 and 2 were high for all biologics (80–100 %). However, groups 5–7 were composed of patients who achieved remission when IFX was administered, while group 8 achieved remission when TCZ or ABT was administered. There were too few patients to make any observations for groups 3 and 4.

## Discussion

Most if not all therapeutic effect prediction studies based on gene expression research have been focused on single rather than multiple biologic agents. Variations in the design of these studies, including recruitment criteria of subjects, evaluation of treatment response, and of assay system platform, represent a huge challenge to combining the studies’ findings in translational studies. Therefore, it is important to develop a unified test platform that allows a level and concomitant comparison among multiple biologic agents and hence anticipation of the therapeutic outcomes. In this study, we have established a clinically practical system to predict the therapeutic effects of three biologics (IFX, TCZ, and ABT). First, we enrolled patients with RA who showed inadequate response to MTX and were administered one of the three biologics for the first time. Second, we used CDAI to evaluate therapeutic effects so as to minimize bias among the three drugs [12, 13]. Third, total RNAs were taken from whole blood with a well-standardized RNA extraction method (PAXgene blood RNA system [14]) and analyzed with a single microarray platform (Agilent Technologies).

There was no overlap of gene sets among the three biologics in GSEA, demonstrating that the molecular targets of each biologic are distinct. This finding encouraged us to proceed with this method for comparing other drugs using this platform.

We observed that NON-REM in the IFX group was typically reflected in upregulated gene expression patterns of the inflammasome, which is a multiprotein complex that plays a key role in the production of inflammatory cytokines, such as proinflammatory cytokines interleukin (IL)-1β and IL-18 [18]. The inflammasome is associated with the pathology of various autoimmune diseases, including RA [19, 20].

Takeuchi et al. reported that the amount of IFX to administer to a patient could be indicated by the baseline TNF protein level to achieve an effective response [21]. Moreover, the inflammasome was reported to be activated downstream of TNF signaling [22–24]. Therefore, our observation of upregulated expression of inflammasome-related genes in the NON-REM group of patients indeed reflected the stimulated TNF signal, which could not be attenuated by a standard amount of IFX. As a result, administering a higher dosage of IFX could be a more plausible approach. Differential expression of TNF mRNA between the REM and NON-REM groups was not observed in our analysis, as TNF protein was found mainly in inflammatory joints rather than in whole blood. In the future, it would be interesting to delineate the relationship between expression of inflammasome-related genes and concentration of TNF in the blood.

For TCZ, we found that a B-cell-related gene set is a promising predictive signature because patients who had a low expression of B cells had poor remission rates. TCZ works as an inhibitor of IL-6 receptor signaling by directly targeting soluble and membrane-bound IL-6 receptors. IL-6 is an important B-cell-stimulating factor and induces antibody synthesis [25], and, in RA pathogenesis, IL-6 induces autoantibody-producing plasma cells [26]. Furthermore, a subset of B cells, especially memory B cells, were previously reported to decrease when TCZ was administered to patients with RA [27, 28]. These findings indicate a close relationship between TCZ response and B cells, as also pointed out by our results. The underlying cause differentiating REM and NON-REM in the TCZ group could be the ability to regulate the amount of B cells and/or the functional subtypes of B cells (memory B cells), as reflected by the expression difference.

NK-cell-related genes were significant predictors of NON-REM in the ABT group. The expression of NK cell-related genes was relatively higher in the NON-REM group than in the REM group. As a component of the innate immune system, NK cells are known to regulate activities of dendritic cells, macrophages and T cells [29]. For example, NK cells were demonstrated to negatively regulate self-responsive T cells in various autoimmune disease models [30–32]. A therapy using ABT, which suppresses T cells, for patients expressing high levels of NK-cell-related genes, which may render activities of T cells suppressed, could be redundant. It is more likely that there are other contributing factors apart from T cells for this type of patient. However, as pointed out by Shegarfi et al. [33], the role of NK cells related to development of RA is worth further delineation.

Core genes found in this study differ from marker genes identified in other studies (IFX: Lequerré et al. [2], Tanino et al. [3], Julia et al. [4], Stuhlmüller et al. [5], Cui et al. [6], and Oswald et al. [34]; TCZ: Sanayama et al. [7]) due to different evaluation parameters of therapeutic outcomes used in each study (e.g., DAS28, EULAR criteria), type of samples used (whole blood or peripheral blood mononuclear cells), and sample size. The most important contributing factor could be the analytical approach. Most biological phenomena, especially development of heterogeneous diseases such as RA, are a consequence not of aberrant individual genes but rather of a network of related genes. Therefore, we employed GSEA to capture the biological feature of genes that would provide a robust model to predict the efficacy of biologics. In fact, functional gene set analysis was successful in identification of interferon gene sets as predictors of the efficacy of rituximab [8, 9].

The performance (i.e., AUCs of ROC curves) of predicting a therapeutic effect (NON-REM) using the signature score for each drug (i.e., inflammasomes, specific CD19, and specific CD56 for IFX, TCZ, and ABT, respectively) were 0.637, 0.796, and 0.768 for IFX, TCZ, and ABT, respectively. At the optimal cutoff value derived from ROC analysis, a notable feature is the high PPVs, which were 83.6 %, 92.3 %, and 94.7 % for IFX, TCZ, and ABT, respectively (Fig. 3). In other words, our approach has a unique feature that could indicate accurately which patients would not likely achieve remission. Although it represents an elimination approach rather than selection of a biologic option, it should be equally effective at a practical clinical level in the context of increasing the probability that a patient would achieve remission. Using this approach, we also discovered a group of patients (Fig. 4, group 1), constituting about 20 % of patients in this study, who were not likely to achieve remission with either biologic (remission rate is a merely 11.9 % [5 of 42]). Future studies exploring biologics other than the three in the present study or differentiation analysis to predict achievement of low disease activity are essential. However, since ROC analysis was conducted using the same sample group that was used to construct the gene signatures, an overfitting problem might occur. It is essential to validate our results in independent cohorts in the future.

While this was an observational study conducted in an actual clinical setting, it also inevitably has limitations, which include bias in clinical background and an unmatched number of samples collected for the studied biologics. In fact, IFX was approved long before TCZ and ABT in Japan, which is self-explanatory why the IFX group outnumbered the TCZ and ABT groups. We believe that the choice of actual clinical settings also led to the bias in clinical background between the three biologics, such as that the ABT group was older than the other two and coadministration with MTX was more likely associated with the IFX group. An independent cohort study in which clinical background is matched could provide a clearer answer. Another limitation was the significant difference in baseline clinical background between the REM and NON-REM groups. Although we have shown that gene expression signature score remains significant after adjusting the baseline clinical background, again, the small number of samples of in the ABT and TCZ groups might not be absolutely persuasive. We are planning to increase the number of samples for validation. Last but not least, as the specimens used in this study contained RNA extracted from whole blood, which is composed of various types of blood cells, it is not clear if the gene expression signatures were just a reflection of different amounts of components of blood cells. This remains to be addressed by analyzing immunophenotyping data in the future.

## Conclusions

We have succeeded in identifying gene expression signatures for prediction of therapeutic effects of three biologics: IFX, TCZ, and ABT. This represents the first attempt in RA treatment history to address three biologics in a unified test platform. The signatures also meet the latest clinical notions regarding the mode of action of each targeted therapy [22, 23, 27, 28]. Therefore, the signatures should not be limited to predicting therapeutic effects but should also provide bases for future studies on prediction of novel drugs as well as a better classification of patients with RA, thus leading to better care.

## Abbreviations

ABT, abatacept; ACPA, anticyclic citrullinated peptide antibodies; ACR, American College of Rheumatology; bDMARD, biologic disease-modifying antirheumatic drug; CDAI, Clinical Disease Activity Index; cRNA, complementary RNA; CRP, C-reactive protein; csDMARD, conventional synthetic disease-modifying antirheumatic drug; Ct, cycle threshold; DAS28, Disease Activity Score in 28 joints; ESR, erythrocyte sedimentation rate; EULAR, European League Against Rheumatism; FDR, false discovery rate; GSEA, Gene Set Enrichment Analysis; IFX, infliximab; LOCF, last observation carried forward; MTX, methotrexate; NK, natural killer; IL, interleukin; NES, normalized enrichment score; NOM, nominal *p* value; NON-REM, patients without Clinical Disease Activity Index remission at 6 months of biologic therapy; NPV, negative predictive value; PhGA, physician global assessment; PPV, positive predictive value; PtGA, patient global assessment; qRT-PCR, real-time quantitative reverse transcription-polymerase chain reaction; RA, rheumatoid arthritis; REM, patients with Clinical Disease Activity Index remission at 6 months of biologic therapy; RF, rheumatoid factor; RNA pol II, RNA polymerase II; SDAI, Simplified Disease Activity Index; SJC28, 28 swollen joint count; TCZ, tocilizumab; TJC28, 28 tender joint count; TNF, tumor necrosis factor.

## Additional files

Additional file 1: Illustration of the distribution of patients in this observational study. (PDF 223 kb) Additional file 2: Blood cell type-specific expressed gene sets. (XLSX 65 kb) Additional file 3: Procedure of signature score calculation. (PDF 202 kb)﻿ Additional file 4: Comparisons of baseline characteristics between REM and NON-REM. (XLSX 17 kb) Additional file 5: Enrichment plots of gene sets extracted from GSEA. (PDF 405 kb) Additional file 6: Ranked gene list by GSEA (IFX analysis). (XLSX 902 kb) Additional file 7: Ranked gene list by GSEA (TCZ analysis). (XLSX 984 kb) Additional file 8: Ranked gene list by GSEA (ABT analysis). (XLSX 1151 kb) Additional file 9: qRT-PCR results of five core genes of each signature identified from GSEA. (PDF 317 kb) Additional file 10: Correlation matrixes of signature scores of core genes for TCZ (**a**) and ABT (**b**). (PDF 3474 kb)
