# Supplementary material for: Identification of baseline gene expression signatures predicting therapeutic responses to three biologic agents in rheumatoid arthritis: a retrospective observational study
Source: Arthritis Res Ther. 2016 Jul 19;18:159. doi: 10.1186/s13075-016-1052-8 (PMC4952232; doi:10.1186/s13075-016-1052-8)
Supplement: Additional file 1: — Illustration of the distribution of patients in this observational study. (PDF 223 kb) [file 13075_2016_1052_MOESM1_ESM.pdf]

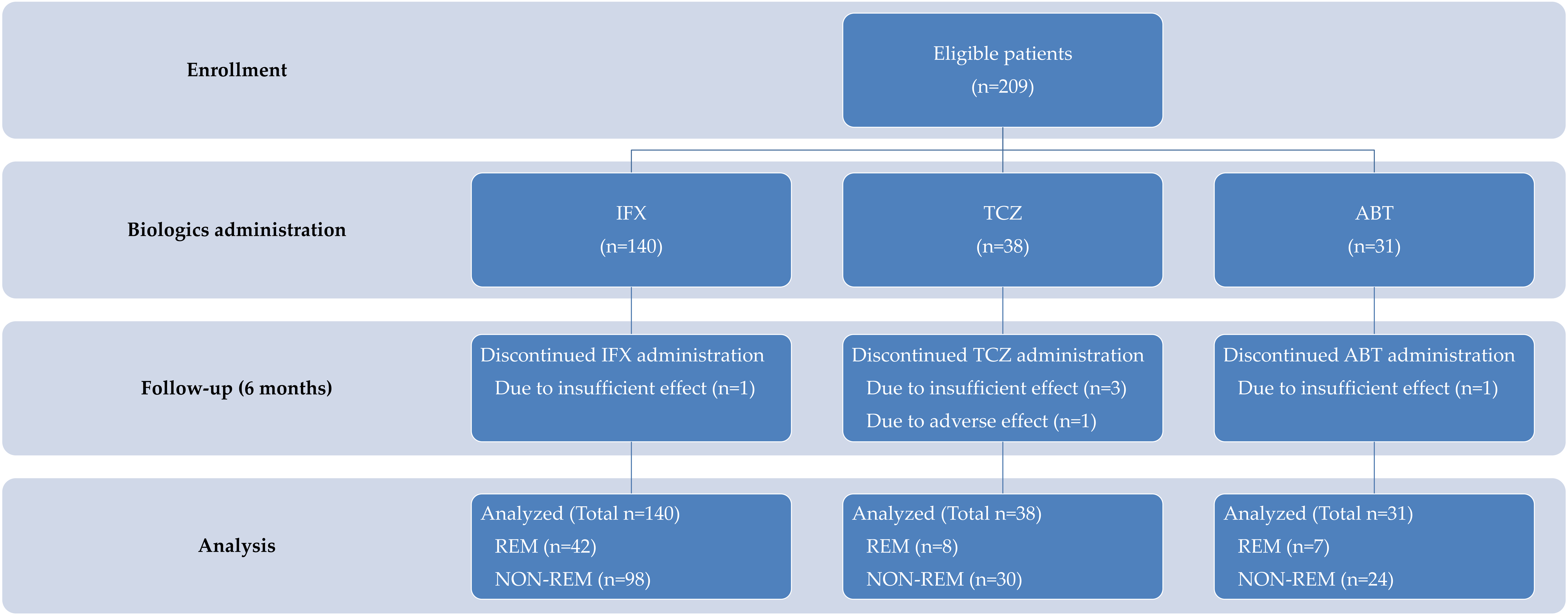

Additional file 4 Illustration of the distribution of patients in this observational study.
IFX, infliximab; TCZ, tocilizumab; ABT, abatacept.
