# Supplementary material for: Identification of baseline gene expression signatures predicting therapeutic responses to three biologic agents in rheumatoid arthritis: a retrospective observational study
Source: Arthritis Res Ther. 2016 Jul 19;18:159. doi: 10.1186/s13075-016-1052-8 (PMC4952232; doi:10.1186/s13075-016-1052-8)
Supplement: Additional file 3: — Procedure of signature score calculation. (PDF 202 kb)﻿ [file 13075_2016_1052_MOESM3_ESM.pdf]

GSEA results

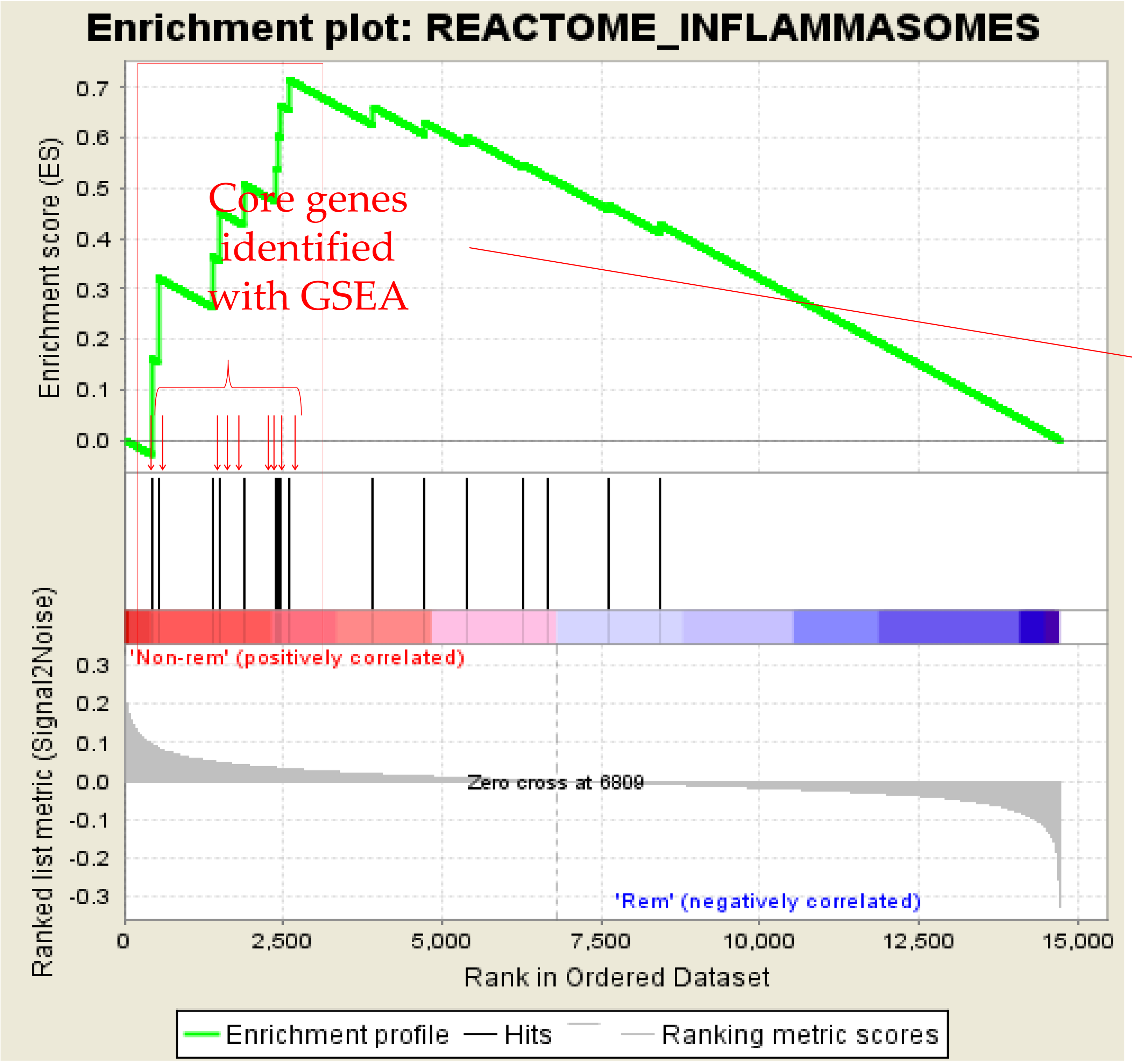

Array data (Quantile normalization values)

Core gene＼Sample	Sample 1	Sample 2	…	Sample 209	Average	S.D.
Core gene 1	9.9	10.2	…	11.3	10.47	0.74
Core gene 2	5.3	4.3	…	2.1	3.90	1.64
Core gene 3	1.1	2.1	…	1.5	1.57	0.50
Core gene 4	5.2	5.5	…	6.2	5.63	0.51
Core gene 5	4.3	4.4	…	5.3	4.67	0.55
Core gene 6	2.3	5.3	…	4.3	3.97	1.53
Core gene 7	4.6	5.8	…	9.8	6.73	2.72
Core gene 8	5.5	7.5	…	3.3	5.43	2.10
Core gene 9	3.2	3.2	…	2.3	2.90	0.52

Z-score transformation across all samples for each gene

Array data (z-score)

Core gene＼Sample	Sample 1	Sample 2	…	Sample 209	Average	S.D.
Core gene 1	-0.77	-0.36	…	1.13	0	1
Core gene 2	0.86	0.24	…	-1.10	0	1
Core gene 3	-0.93	1.06	…	-0.13	0	1
Core gene 4	-0.84	-0.26	…	1.10	0	1
Core gene 5	-0.67	-0.48	…	1.15	0	1
Core gene 6	-1.09	0.87	…	0.22	0	1
Core gene 7	-0.78	-0.34	…	1.13	0	1
Core gene 8	0.03	0.98	…	-1.02	0	1
Core gene 9	0.58	0.58	…	-1.15	0	1

Averaging z-score across all core genes for each sample

Signature score data

Core gene＼Sample	Sample 1	Sample 2	…	Sample 209
Signature score	-0.40	0.25	…	0.15

Additional file 2 Procedure of signature score calculation. First, we extracted significant core genes from GSEA. Then each core gene was standardized using a z-score transformation method based on 209 patients' data. The average z-score of the core genes was defined as a signature score of the target gene set for each patient.
