# Supplementary material for: Identification of baseline gene expression signatures predicting therapeutic responses to three biologic agents in rheumatoid arthritis: a retrospective observational study
Source: Arthritis Res Ther. 2016 Jul 19;18:159. doi: 10.1186/s13075-016-1052-8 (PMC4952232; doi:10.1186/s13075-016-1052-8)
Supplement: Additional file 5: — Enrichment plots of gene sets extracted from GSEA. (PDF 405 kb) [file 13075_2016_1052_MOESM5_ESM.pdf]

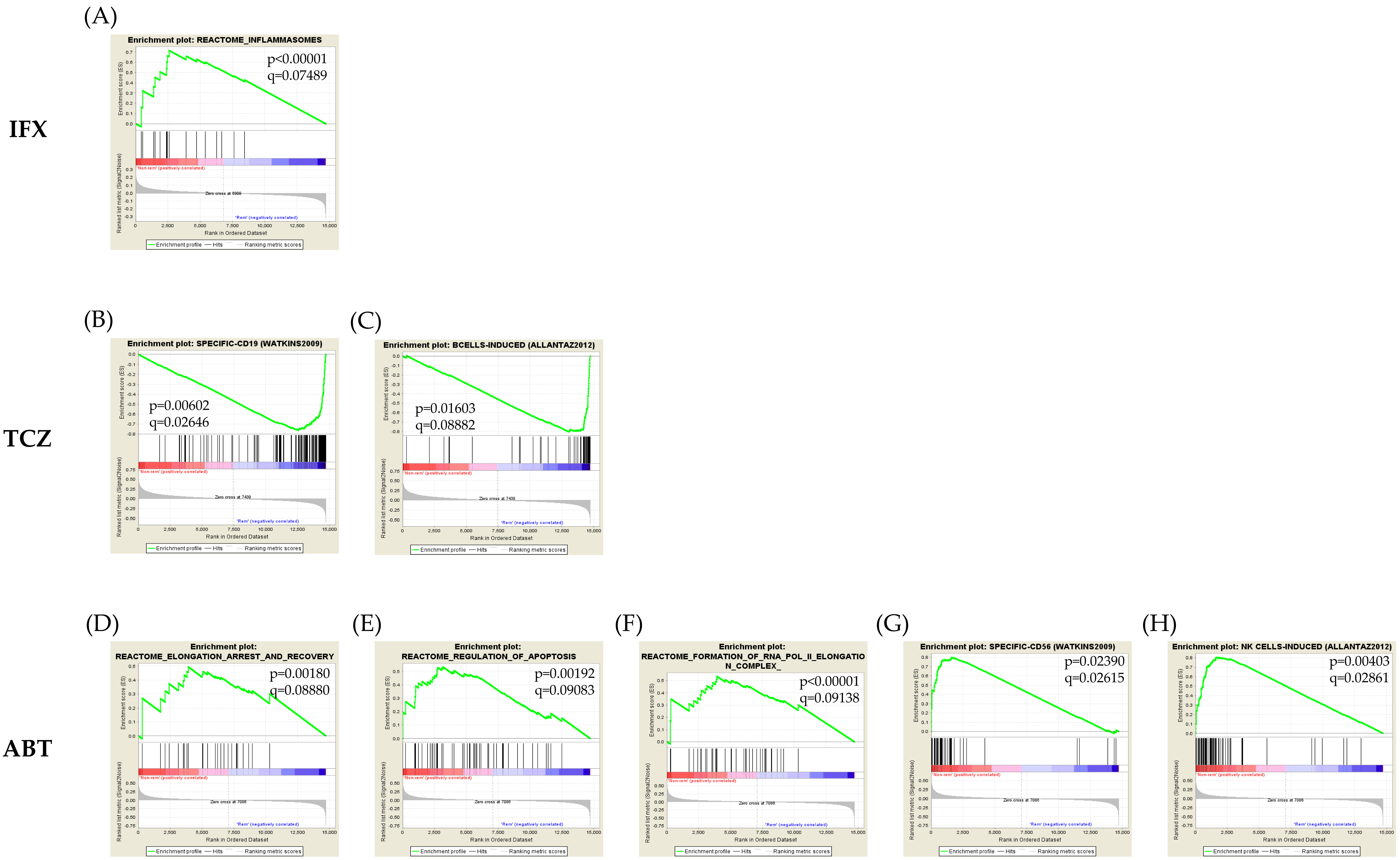

Additional file 5 Enrichment-plots of gene sets extracted from GSEA. (A) inflammasomes, (B) Specific-CD19 (Watkins 2009), (C) B cells-induced (Allantaz 2012), (D) Elongation arrest and recovery, (E) Regulation of apoptosis, (F) Formation of RNA pol II elongation complex, (G) Specific-CD56 (Watkins 2009), (H) NK cells-induced (Allantaz 2012).
A p-value < 0.05 and a q-value < 0.1 was considered statistically significant.
REM, patients with CDAI remission* at 6 months of biologic therapy; NON-REM, patients without CDAI remission* at 6 months of biologic therapy.
* CDAI remission is defined as CDAI <= 2.8.
