# Supplementary material for: Identification of baseline gene expression signatures predicting therapeutic responses to three biologic agents in rheumatoid arthritis: a retrospective observational study
Source: Arthritis Res Ther. 2016 Jul 19;18:159. doi: 10.1186/s13075-016-1052-8 (PMC4952232; doi:10.1186/s13075-016-1052-8)
Supplement: Additional file 9: — qRT-PCR results of five core genes of each signature identified from GSEA. (PDF 317 kb) [file 13075_2016_1052_MOESM9_ESM.pdf]

Inflammasomes

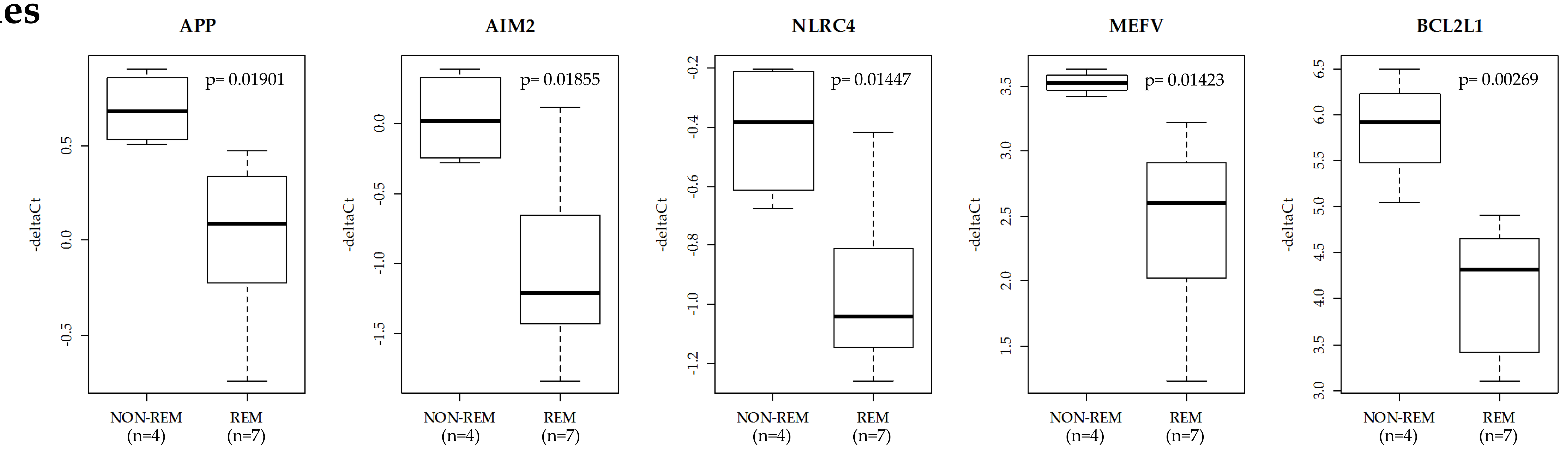

Specific-CD19

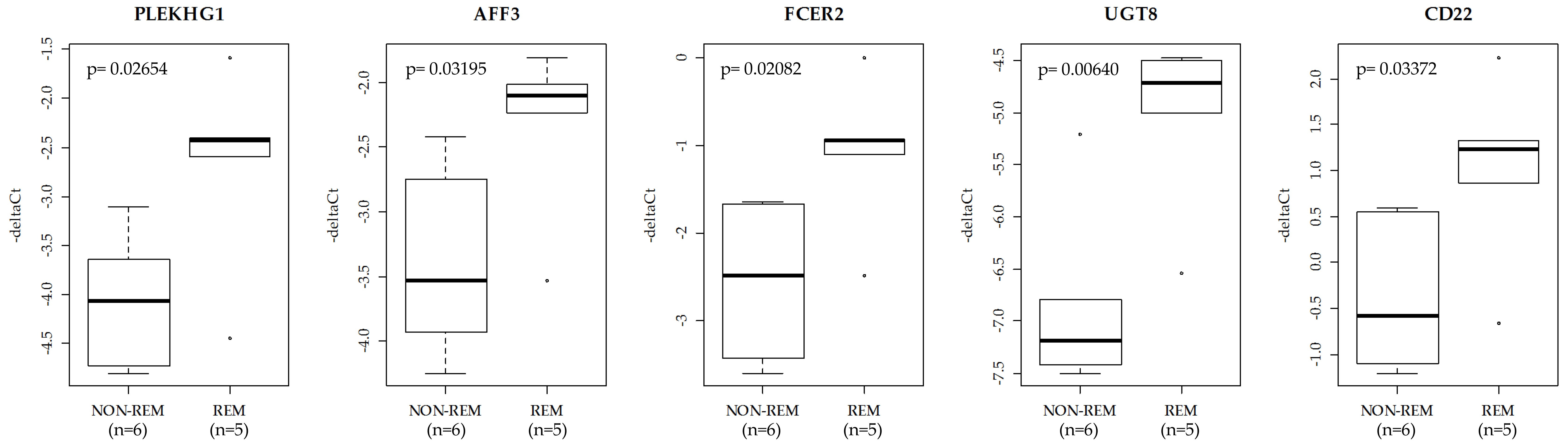

Specific-CD56

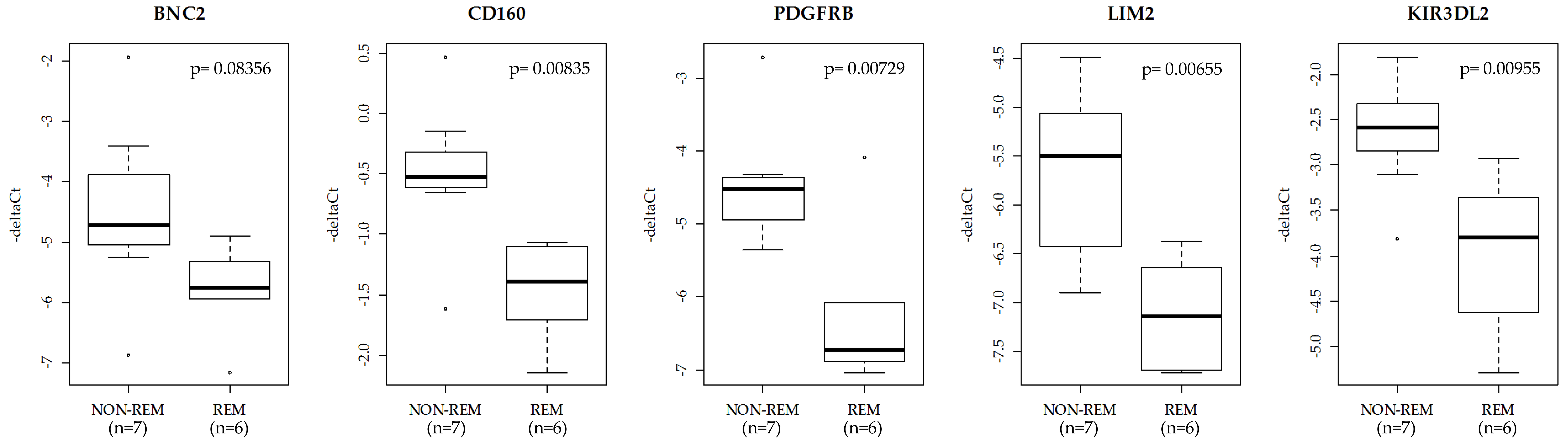

Additional file 9 qRT-PCR results of 5 core genes of each signature identified from GSEA. GUSB was used as an internal control to normalize cDNA input. Distribution of –deltaCt values within REM and NON-REM were compared using boxplots. p-Values were determined using Student's t-test.
REM, patients with CDAI remission* at 6 months of biologic therapy; NON-REM, patients without CDAI remission* at 6 months of biologic therapy.
* CDAI remission is defined as CDAI <= 2.8.
