# Supplementary material for: Identification of baseline gene expression signatures predicting therapeutic responses to three biologic agents in rheumatoid arthritis: a retrospective observational study
Source: Arthritis Res Ther. 2016 Jul 19;18:159. doi: 10.1186/s13075-016-1052-8 (PMC4952232; doi:10.1186/s13075-016-1052-8)
Supplement: Additional file 10: — Correlation matrixes of signature scores of core genes for TCZ (a) and ABT (b). (PDF 3474 kb) [file 13075_2016_1052_MOESM10_ESM.pdf]

Arthritis Research & Therapy

Identification of baseline gene expression signatures predicting therapeutic responses to three biologic agents in rheumatoid arthritis: a retrospective observational study

--Manuscript Draft--

**Manuscript Number:**	ARRT-D-16-00110R2
**Full Title:**	Identification of baseline gene expression signatures predicting therapeutic responses to three biologic agents in rheumatoid arthritis: a retrospective observational study
**Article Type:**	Research article
**Section/Category:**	Genetics and Epigenetics
**Funding Information:**	
**Abstract:**	Background<br>According to EULAR recommendations, biological DMARDs (bDMARDs) such as tumor necrosis factor inhibitor (TNFi), tocilizumab (TCZ), and abatacept (ABT) are in parallel when prescribing to rheumatoid arthritis (RA) patients who have shown insufficient response to conventional synthetic DMARDs. However, most prediction studies of therapeutic response of bDMARDs using gene expression profiles focused on only single bDMARD, and considerations of the results from the perspective of RA pathophysiology were not sufficient. The aim of this study was to identify the specific molecular biological features predicting the therapeutic outcomes of three bDMARDs, infliximab (IFX), TCZ, and ABT by studying blood gene expression signature of patients prior to biologic treatment in a unified test platform.<br><br>Methods<br>RA patients who responded inadequately to methotrexate and were later commenced with any one of IFX (n=140), TCZ (n=38), and ABT (n=31) as their first biologic between May 2007 and November 2011 were enrolled. Whole blood gene expression data were obtained prior to their biologic administration. They were defined as remission; "REM" and non-remission; "NON-REM" groups, according to CDAI at 6 months of biologic therapy. We employed Gene Set Enrichment Analysis (GSEA) to identify functional gene sets differentially expressed between these 2 groups for each biologic. Then, we compiled "signature scores" for these gene sets and the prediction performances were assessed.<br><br>Results<br>GSEA analyses showed that inflammasome genes were significantly upregulated in IFX's NON-REM compared with its REM. In TCZ's REM, B cell-specifically expressed genes were upregulated. RNA elongation, apoptosis-related, and NK cell-specifically expressed genes were upregulated in ABT's NON-REM. Logistic regression analyses showed "signature scores" of inflammasomes, B cell-specifically expressed, and NK cell-specifically expressed genes were significant independent predictive factors for treatment outcome with IFX, TCZ, and ABT, respectively. The AUCs of ROC curves of these signature scores were 0.637, 0.796, and 0.768 for IFX, TCZ, and ABT, respectively.<br><br>Conclusions<br>We have identified original gene expression predictive signatures uniquely underlying the therapeutic effects of IFX, TCZ and ABT. This is, known by far the first attempt to predict therapeutic effects of three drugs concomitantly using a unified gene expression test platform.
**Corresponding Author:**	Seiji Nakamura<br>DNA Chip Research Inc.<br>Minato-ku, Tokyo JAPAN
**Corresponding Author Secondary Information:**	
**Corresponding Author's Institution:**	DNA Chip Research Inc.

Powered by Editorial Manager® and ProduXion Manager® from Aries Systems Corporation

Corresponding Author's Secondary Institution:	
First Author:	Seiji Nakamura
First Author Secondary Information:	
Order of Authors:	Seiji Nakamura
	Katsuya Suzuki
	Hiroshi Iijima
	Yuko Hata
	Chun Ren Lim
	Yohei Ishizawa
	Hideto Kameda
	Koichi Amano
	Kenichi Matsubara
	Ryo Matoba
	Tsutomu Takeuchi
Order of Authors Secondary Information:	
Response to Reviewers:	RESPONSE TO REVIEWER #1:<br><br>--<br>Point 1: Although the new text addresses the point that I raised, the text is difficult to understand. Specific details are: the reference to RA as a malignancy, the meaning of "standards used to predict efficacy", and of "total number of 2 cohorts analyzed..." Other sentences that are difficult to understand are those making reference to Oswald et al., and mentioning different approaches for analysis.<br><br>Response: By referring to Oswald et al., (an important paper recommended by reviewer #2) at the end of the paragraph, we have incorporated expressions that require explanations. We have therefore moved the reference to the beginning of the paragraph so that it is presented together with other references of previous studies addressing similar topic. Furthermore, we have also rephrased the expressions above.<br><br>Before revision<br>Core genes found in this study differ from other studies (TNFi: Lequerre et al [2], Tanino et al [3], Julia et al [4], Stuhlmuller et al [5], Cui et al [6], TCZ: Sanayama et al [7]) mainly due to the standards used to predict efficacy, type of samples (whole blood or peripheral mononuclear cells) and number of samples used. The most contributing factor could be the analysis approach. Most biological phenomena especially development of a malignancy as heterogeneous as RA, are not a consequence of aberrant individual genes but rather a network of related genes. Therefore we have employed GSEA to capture the feature of genes that will provide a robust model to predict the efficacy of biologics. In fact, while reproducibility was poor using individual genes approach, functional gene set analysis was successful in identification of interferon gene sets as predictor of efficacy of rituximab [8-9]. On the other hand, modular expression approach by Oswald et al was not able to capture gene modules that predict therapeutic effects of different drugs of TNFi [33]. Differences in recruitment criteria of samples (whether they were first biologic and MTX-failed) and also evaluation standards of treatment outcome render direct comparison with current study difficult. In addition, total number of 2 cohorts analyzed for IFX (n=43), the only biologic shared with this study, is arguably smaller than the number of current study (n=140).<br><br>After revision (p. 19, line 20 - p. 20, line 9)<br>Core genes found in this study differ from marker genes identified in other studies (IFX: Lequerre et al [2], Tanino et al [3], Julia et al [4], Stuhlmuller et al [5], Cui et al [6], Oswald et al [34], TCZ: Sanayama et al [7]) due to different evaluation parameters of

Powered by Editorial Manager® and ProduXion Manager® from Aries Systems Corporation

therapeutic outcome used in each study (DAS28, EULAR criteria etc.), type of samples (whole blood or PMBC) and sample size. The most contributing factor could be the analysis approach. Most biological phenomena especially development of heterogeneous disease like RA, are not a consequence of aberrant individual genes but rather a network of related genes. Therefore we have employed GSEA to capture the biological feature of genes that will provide a robust model to predict the efficacy of biologics. In fact, functional gene set analysis was successful in identification of interferon gene sets as predictor of efficacy of rituximab [8-9].

Point 2: I fear my comment on this point was not clear. What I intended was to request information on the criteria used to treat some of the patients with Tocilizumab and Abatacept. This needs explanation because none of these two drugs was a first line biologic at the time the patients were treated.

Response: Administration of each biologic is based on JCR guidelines (http://www.ryumachi-jp.com/guideline.html). We have revised the sentences to deliver the points accordingly.

Before revision

Eligible patients were those who met the 1987 revised criteria of the American College of Rheumatology (ACR) for the classification of RA or the 2010 ACR/EULAR classification criteria and responded inadequately (clinical disease activity index: CDAI > 2.8) to MTX (> 6mg/week). RA patients who were commenced with any one of IFX, TCZ, and ABT as their first biologic between May 2007 and November 2011 at Keio University Hospital and Saitama Medical University Saitama Medical Center were enrolled. IFX, TCZ, and ABT were approved as first line biologics in Japan in 2003, 2008 and 2010, respectively and selection was based on Japanese clinical guidelines in parallel with physician's judgement.

After revision (p. 7, line 3 - 11)

The diagnosis of RA was based on the 1987 revised criteria of the American College of Rheumatology (ACR) for the classification of RA or the 2010 ACR/EULAR classification criteria. RA patients who responded inadequately to MTX (>= 6mg/week) and were commenced with any one of IFX, TCZ (2008-), and ABT (2010-) as their first biologic between May 2007 and November 2011 at Keio University Hospital and Saitama Medical University Saitama Medical Center were enrolled. Biologics were administered according to the guidelines set by Japan College of Rheumatology (JCR) (http://www.ryumachi-jp.com/guideline.html).

Point 13: Quantile normalization does not protect against batch effects. Similarly, validation of top findings is not sufficient warranty. I think a satisfactory evidence would be negative results in any method applied to check for batch effects like cluster analysis or PCA. If batch effects are present, they should be corrected. The most common approach is to use Combat.

Response: Please find figure (Cover letter_revised.docx) of Hierarchical clustering of samples after Quantile normalization.

Microarray experiment of current study was performed accordingly as blood samples of study subjects were recruited from year 2008 to 2013. In the figure, we have therefore color-labeled the samples according to the year when data were obtained. Cluster analysis results are often dependent on the algorithms used and hence we have applied 2 types of algorithms, i.e. A Similarity Measure: Euclidean, Linkage Rule: Centroid, B Similarity Measure: Euclidean, Linkage Rule: Complete, to find if results are consistent.

No particular cluster was observed according to the timing when experiments were conducted for both algorithms and this leads us to conclude that batch effects are negligible. Furthermore, as shown in the previous revision, the signatures obtained from microarray data are reproducible using another methodology, qPCR, and this should solidify the features of signatures we obtained in this study.

Powered by Editorial Manager® and ProduXion Manager® from Aries Systems Corporation

	Point 17: The paragraph on discussion about NK cells and CD86 in the response to Abatacept is very speculative and I think it should be deleted.<br><br>Response: Thank you for your advice. We have replaced the discussion for NK cells as below.<br><br>Before revision<br>NK cell-related genes comprise significant predictors of ABT's NON-REM: the expression of NK cell-related genes is relatively higher in NON-REM than that of REM. NK cells have a close relationship with CD86, the target of ABT. IFNg from NK cells or NK cells themselves are responsible for the maturing of dendritic cells which express CD86 [29-31]. NK cells were also reported to express CD86 [32]. The up-regulated expression pattern of NK cells gene sets and IFNg gene (Additional file 8) could be related to activation of NK cells or increase in the number of NK cells, which trigger excessive production of CD86. This may lead to insufficient amount of ABT that has been administered to patients with up-regulated expression pattern of NK cells, to suppress disease activity. The roles of NK cells in the physiological pathology of RA are certainly a field awaiting for more research.<br><br>After revision (p. 19, line 8 - 19)<br>NK cell-related genes comprise significant predictors of ABT's NON-REM: the expression of NK cell-related genes is relatively higher in NON-REM than that of REM. As a component of innate immune system, NK cells are known to regulate activities of dendritic cells, macrophages and T cells [29]. For example, NK cells were demonstrated to negatively regulate self-responsive T cells in various autoimmune disease models [30-32]. A therapy using ABT, which suppresses T cell, to patients expressing high level of NK cell-related genes, which may render activities of T cell being suppressed, could be redundant. It is more likely that there are other contributing factors apart from T cells for this type of patient. However, as pointed out by Shegarfi et al [33], the role of NK cells related to development of RA should worth further delineation.<br>--

Powered by Editorial Manager® and ProduXion Manager® from Aries Systems Corporation

Manuscript_revised
Click here to download Manuscript Manuscript revised.docx
Click here to view linked References

Identification of baseline gene expression signatures predicting therapeutic responses to three biologic agents in rheumatoid arthritis: a retrospective observational study

Authors

Seiji Nakamura **(corresponding and submitting author)**
DNA Chip Research Inc.
1-15-1 Kaigan, Suzuebaydium 5F, Minato-ku, Tokyo 105-0022, Japan
E-mail: nakamura@dna-chip.co.jp
Tel: + 81-3-5777-1700
Fax: + 81-3-1689-1687

Katsuya Suzuki
Division of Rheumatology, Department of Internal Medicine, Keio University School of Medicine
35 Shinanomachi, Shinjuku-ku, Tokyo 160-8582, Japan
E-mail: katsuyas@z5.keio.jp

Hiroshi Iijima
DNA Chip Research Inc.
1-15-1 Kaigan, Suzuebaydium 5F, Minato-ku, Tokyo 105-0022, Japan
E-mail: h-iijima@dna-chip.co.jp

Yuko Hata
DNA Chip Research Inc.
1-15-1 Kaigan, Suzuebaydium 5F, Minato-ku, Tokyo 105-0022, Japan
E-mail: hata@dna-chip.co.jp

Chun Ren Lim
DNA Chip Research Inc.
1-15-1 Kaigan, Suzuebaydium 5F, Minato-ku, Tokyo 105-0022, Japan
E-mail: cr-lim@dna-chip.co.jp

Yohei Ishizawa
DNA Chip Research Inc.
1-15-1 Kaigan, Suzuebaydium 5F, Minato-ku, Tokyo 105-0022, Japan
E-mail: ishizawa@dna-chip.co.jp

Hideto Kameda
Division of Rheumatology, Department of Internal Medicine, Toho University Ohashi Medical Center
2-17-6, Ohashi Muguro-ku, Tokyo, 153-8515, Japan
E-mail: hideto.kameda@med.toho-u.ac.jp

Koichi Amano
Department of Rheumatology and Clinical Immunology, Saitama Medical Center, Saitama Medical University
1981 Tsujido-machi Kamoda, Kawagoe-shi, Saitama, 350-8550, Japan
E-mail: amanokoi@saitama-med.ac.jp

Kenichi Matsubara
DNA Chip Research Inc.
1-15-1 Kaigan, Suzuebaydium 5F, Minato-ku, Tokyo 105-0022, Japan
E-mail: matsubara@dna-chip.co.jp

Ryo Matoba
DNA Chip Research Inc.
1-15-1 Kaigan, Suzuebaydium 5F, Minato-ku, Tokyo 105-0022, Japan
E-mail: matoba@dna-chip.co.jp

Tsutomu Takeuchi
Division of Rheumatology, Department of Internal Medicine, Keio University School of Medicine
35 Shinanomachi, Shinjuku-ku, Tokyo 160-8582, Japan
E-mail: tsutake@keio.jp

Abstract

Background

According to EULAR recommendations, biological DMARDs (bDMARDs) such as tumor necrosis factor inhibitor (TNFi), tocilizumab (TCZ), and abatacept (ABT) are in parallel when prescribing to rheumatoid arthritis (RA) patients who have shown insufficient response to conventional synthetic DMARDs. However, most prediction studies of therapeutic response of bDMARDs using gene expression profiles focused on only single bDMARD, and considerations of the results from the perspective of RA pathophysiology were not sufficient. The aim of this study was to identify the specific molecular biological features predicting the therapeutic outcomes of three bDMARDs, infliximab (IFX), TCZ, and ABT by studying blood gene expression signature of patients prior to biologic treatment in a unified test platform.

Methods

RA patients who responded inadequately to methotrexate and were later commenced with any one of IFX (n=140), TCZ (n=38), and ABT (n=31) as their first biologic between May 2007 and November 2011 were enrolled. Whole blood gene expression data were obtained prior to their biologic administration. They were defined as remission; "REM" and non-remission; "NON-REM" groups, according to CDAI at 6 months of biologic therapy. We employed Gene Set Enrichment Analysis (GSEA) to identify functional gene sets differentially

expressed between these 2 groups for each biologic. Then, we compiled "signature scores" for these gene sets and the prediction performances were assessed.

Results

GSEA analyses showed that inflammasome genes were significantly upregulated in IFX's NON-REM compared with its REM. In TCZ's REM, B cell-specifically expressed genes were upregulated. RNA elongation, apoptosis-related, and NK cell-specifically expressed genes were upregulated in ABT's NON-REM. Logistic regression analyses showed "signature scores" of inflammasomes, B cell-specifically expressed, and NK cell-specifically expressed genes were significant independent predictive factors for treatment outcome with IFX, TCZ, and ABT, respectively. The AUCs of ROC curves of these signature scores were 0.637, 0.796, and 0.768 for IFX, TCZ, and ABT, respectively.

Conclusions

We have identified original gene expression predictive signatures uniquely underlying the therapeutic effects of IFX, TCZ and ABT. This is, known by far the first attempt to predict therapeutic effects of three drugs concomitantly using a unified gene expression test platform.

Keywords

Rheumatoid arthritis, bDMARDs (Biologic agent), Prediction, Gene expression

Introduction

Methotrexate (MTX) and biological disease-modifying anti-rheumatic drugs (bDMARDs) have brought therapeutic successes to most rheumatoid arthritis (RA) patients but not all. Optimization of treatment for individual patient and development of novel therapies are eagerly anticipated. A good effort of the former is to establish standard methodology to determine which bDMARDs to prescribe. As molecular target of each bDMARD is distinct, each effective treatment should link to changes in a/several particular biological process/es which are ultimately manifested as disease state. Despite the understanding, evidence supporting current concepts of prescription of bDMARDs is unsatisfactory. According to the European League Against Rheumatism (EULAR) recommendations, tumor necrosis factor inhibitor (TNFi), tocilizumab (TCZ), and abatacept (ABT) are in parallel when determining first biologics for RA patients who have shown inadequate response using conventional synthetic DMARDs (csDMARDs) [1]. Development of a methodology to determine effective therapy using bDMARDs is definitely essential.

Most prediction studies of therapeutic response of bDMARDs using gene expression profiles of blood samples focused on single biologic [2-9] and to date no report of multiple drugs studied in parallel is available. Furthermore, study designs varied and thus render translational study very challenging.

Difficulty to reproduce gene expression studies has also plagued the situation, partly due to non-uniformities of study design but also data

processing itself [10]. Instead of incorporating existing biological knowledge, analysis rarely extends beyond individual gene-level to explain how the findings of biomarkers are associated with the mode of actions related to targeted therapies of RA. Furthermore, to establish a robust model using gene expression, it is essential to interpret the result as effects of a collective network of related genes rather than that of gene per se. In this context, Gene Set Enrichment Analysis (GSEA) [11], which was shown to detect differentially-expressed functional gene sets, should be a promising approach.

In this study, to identify therapeutic efficacy markers of three bDMARDs (IFX, TCZ, and ABT) targeting at different molecules, we take the aforementioned problems into considerations. We designed “a unified test platform” where recruitment criteria of subjects, evaluation of treatment response and platform of assay system are well defined. GSEA is employed to identify and annotate the gene signatures associated with each biologic. The prediction performance, biological interpretation and utility of each gene signature are presented.

Methods

Patients and evaluation of effectiveness

The diagnosis of RA was based on the 1987 revised criteria of the American College of Rheumatology (ACR) for the classification of RA or the 2010 ACR/EULAR classification criteria. RA patients who responded inadequately to MTX (>= 6mg/week) and were commenced with any one of IFX, TCZ (2008-), and ABT (2010-) as their first biologic between May 2007 and November 2011 at Keio University Hospital and Saitama Medical University Saitama Medical Center were enrolled. Biologics were administered according to the guidelines set by Japan College of Rheumatology (JCR) (http://www.ryumachi-jp.com/guideline.html). Therapeutic outcomes were defined as achieving remission "REM" (defined as CDAI <= 2.8) or not achieving remission "NON-REM" using CDAI at 6 months of biologic therapy, since other disease activity indexes such as DAS28 etc. incorporate inflammatory factors like CRP, ESR, which may overestimate the efficacy of TCZ [12-13]. Patients who discontinued biologic therapy by 6 months due to insufficient effects (n=5) or adverse events (n=1) were classified as "NON-REM". CDAI of all 6 cases were more than 2.8 as determined using last observation carried forward (LOCF). Written informed consents were obtained from all patients in accordance with the Helsinki protocol and the study protocol was approved by the institutional review boards at Keio University and Saitama Medical University.

RNA extraction

Before administration with biologic agent, blood samples were collected in PAXgene Blood RNA tubes [14] (PreAnalytiX, Hombrechtikon, Switzerland). Total RNAs were extracted using PAXgene Blood RNA Kits (PreAnalytiX) following the manufacturer's instructions. Total RNA quantity and quality were determined using a NanoDrop-1000 spectrophotometer (Thermo Fisher Scientific, Waltham, MA, USA) and an Agilent 2100 Bioanalyzer (Agilent Technologies, Palo Alto, CA, USA). All RNA samples fulfilled both criteria as followed: RNA integrity number > 6.5, and OD260/280 > 1.6.

Gene expression measurements

Cy3-labeled complementary RNAs (cRNAs) were synthesized using Quick Amp Labeling Kits (Agilent). The cRNAs were hybridized at 65°C for 17 hours to Whole Human Genome 44K Microarrays (Agilent, Design ID: 014850). After washing, the microarrays were scanned using an Agilent DNA microarray scanner (Agilent). Intensity values of each scanned feature were quantified using Agilent Feature Extraction Software (Agilent). The raw microarray data are deposited in the National Center for Biotechnology Information Gene Expression Omnibus (accession number: GSE78068). We applied rank-based quantile normalization to the raw signal data using R software version 3.0.2. Next, probes were filtered based on pre-existing annotation with gene symbol

and signal intensity (called "Present" in more than 50 samples according to Agilent GeneSpring Software). For genes with more than one probe, we adopted the probe that has the highest signal intensity. Final number of probes for subsequent analysis was 14,718.

GSEA analysis

We employed GSEA to study the molecular biological features associated with REM and NON-REM groups of each biologic therapy. GSEA is a computational method that determines whether an a priori defined set of genes shows statistically significant, concordant differences between two biological states [11].

We used GSEA v2.1.0 and input data were 3 sets of data matrics, i.e. 14718 genes × 140 samples (IFX), 14718 genes × 38 samples (TCZ), 14718 genes × 31 samples (ABT). Two lists of gene sets were used as a priori defined sets of genes, i.e. Reactome gene sets at MsigDB repository [11], containing 674 pathways and integrated lists of blood cell type-specific expressed gene sets by Watkins et al. and Allantaz et al [15-16]. The integrated lists have 16 blood cell type-specific expressed gene sets (Additional file 1). Permutation type was set as "phenotype" and number of permutation is 1000. The population gene set for analysis was 14,718 and metrics for ranking genes were in Signal2Noise ratio. Gene set size filters were in default settings where min=15 and max=500. Gene sets with a nominal p-value < 0.05 and false discovery rate < 0.1 were

considered significant. Then, we defined “core genes” as the subset of genes that contributed most to the GSEA enrichment score.

Real-Time quantitative reverse transcription PCR (qRT-PCR)

qRT-PCR was performed for 11-13 samples of each biologic where total RNA’s were adequate. Genes measured were APP, AIM2, NLRC4, MEFV, BCL2L1 for “inflammasomes” (signature of IFX), PLEKHG1, AFF3, FCER2, UGT8, CD22 for "specific-CD19" (signature of TCZ), and BNC2, CD160, PDGFRB, LIM2, KIR3DL2 for "specific-CD56" (signature of ABT). We designed custom RT^2 Profiler PCR Arrays (QIAGEN, Valencia, CA, USA) and assay was performed according to manufacturer's instructions. Essentially, 500ng of total RNA of each sample was used to synthesize cDNA using RT^2 HT First Strand Kit (QIAGEN). qRT-PCR was performed using Applied Biosystems 7500 Fast Dx Real-time PCR System (Thermo Fisher Scientific). Relative expression value of each probe (-deltaCt value) was derived against the Ct of internal control GUSB.

Calculation of signature score

A scoring system, which is clinically applicable to each patient, was shown in Additional file 2. Briefly, each core gene that belonged to a target gene set was standardized using a z-score transformation based on all 209 patients' data, and then the average of z-scores of all core genes was defined as the “signature score” of the gene set for each patient.

Receiver operating characteristic (ROC) analysis

ROC analysis was conducted using signature score compared against REM-versus-NON-REM and area under the ROC curve (AUC) was determined. We applied the same sample group that was used to construct gene signature. NON-REM was defined as “Positive”. Sensitivity, specificity, positive predictive value (PPV) and negative predictive value (NPV) were determined at the optimal cut-off value (threshold) from ROC. Analysis was performed using R software version 3.0.2.

Statistics

CDAI of 6 samples (Additional file 4) where administration was terminated before 6 months, were estimated using LOCF. Kruskal–Wallis test, Wilcoxon's rank sum test, or Student's t-test was performed for numerical variables. For categorical variables, Fisher's exact test was conducted. The associations between CDAI remission at 6 months of biologic therapy and signature scores were evaluated using univariate and multivariate logistic regression analyses (Firth’s penalized likelihood method [17]). For multivariate analyses, we adjusted for marginally significant ($p < 0.1$) univariate factors as in Additional file 3, but TJC28, SJC28, PtGA, PhGA, DAS28-ESR, and SDAI in IFX analysis, SJC28, DAS28-ESR, and SDAI in TCZ, concomitant steroid dose in ABT were not adjusted due to the strong correlation with CDAI (in IFX and TCZ analysis) or concomitant steroid use (in ABT analysis).

In this study, a p-value of < 0.05 was considered significant. p-Values from these analyses were not adjusted for multiple testing. All statistical analyses were performed with R software version 3.0.2.

Results

Baseline clinical characteristics and therapeutic response to each biologic therapy

There are 140, 38 and 31 cases (total 209) of IFX, TCZ and ABT, respectively. Baseline characteristics of the enrolled patients of 3 biologic groups are shown in Table 1. Median of age of 209 samples was 59 years old and disease duration was 3.3 years. Co-administration of MTX had a median volume of 8mg and the median of CDAI was 21.7. Among the biologic agents, ABT group was consisted of an older age group of subjects and co-administration of MTX was slightly higher in IFX group.

Administration was terminated by 6 months for IFX (n=1), TCZ (n=3) and ABT (n=1) due to insufficient effect (Additional file 4). And there was an adverse effect case in TCZ. These cases were subsequently classified as “NON-REM”. For all 209 cases, 27.3% achieved remission at 6 months of biologic therapy (Figure 1). The remission rates of IFX, TCZ and ABT were 30.0%, 21.1% and 22.6%, respectively.

Differences in baseline characteristics between REM and NON-REM are shown in Additional file 3. There were significant differences in female proportion (p=0.034) and concomitant use of csDMARDs besides MTX (p=0.016) between REM and NON-REM for IFX. SJC28, PtGA, PhGA, DAS28ESR, SDAI, CDAI were also significantly higher in IFX’s NON-REM. For TCZ, NON-REM had a higher DAS28ESR (p=0.045). The duration of disease for ABT group was

longer in NON-REM (p=0.003).

Baseline gene expression features underlying REM and NON-REM

We conducted GSEA analyses to identify the molecular biological features underlying REM and NON-REM groups of each biologic therapy. It is a powerful analytical method to detect modest but coordinated changes in the expression of groups of functionally related genes. Table 2 summarizes the results of GSEA (see also Additional file 5-8). In Reactome gene sets analyses, “inflammasomes” in IFX, "elongation arrest and recovery", "regulation of apoptosis" and "formation of RNA pol II elongation complex" in ABT, show up-regulated expression patterns for NON-REM. In blood cell gene sets analysis, signature related to B cells, such as "specific-CD19" and "B cells-induced" showed up-regulated expression patterns in TCZ for REM. In ABT, signature related to natural killer (NK) cells, such as "specific-CD56" and "NK cells-induced" showed up-regulated expression patterns for NON-REM. Top performing genes for each gene set were validated using qRT-PCR (Additional file 9).

Signature scores and therapeutic responses

We compiled “signature scores” based on gene sets identified using GSEA, to evaluate individual gene expression profiles (see Methods). Signature scores generated were able to significantly differentiate REM and NON-REM

(Figure 2A-H) and thus could serve as a system to predict each individual's prospective therapeutic outcome.

We found overlapping genes especially within gene sets of TCZ and within gene sets of ABT (Additional file 7 and 8). Correlation analysis of signature scores of these gene sets indeed confirmed redundancy (Additional file 9). For predictive signature of TCZ, ″specific-CD19″ and ″B cells-induced″ were consolidated as ″specific-CD19″ (Pearson's correlation coefficient=0.99). For ABT, "elongation arrest and recovery", "regulation of apoptosis" , "formation of RNA pol II elongation complex" were closely related (correlation coefficient=0.77, 0.91, 0.84) while "specific-CD56" ,"NK cells-induced" also shared a high correlation (correlation coefficient=0.96). We thus subsequently focused on ″elongation arrest and recovery″ and ″specific-CD56″.

Logistic regression analyses of signature scores as univariate independent variable and CDAI remission as dependent variable, showed each signature score was significant (Table 3). In multivariate analyses where clinical background of REM and NON-REM (Table 1) were also taken into account, signature scores remained significant. We also found "specific-CD56" was more significant compared to "elongation arrest and recovery" in ABT, and thus concentrated on "specific-CD56" for subsequent analysis.

Evaluation of the prediction performance using signature score

ROC analysis was performed using signature scores (Figure 3). The AUCs of

signature scores to predict NON-REM based on CDAI were 0.637 (IFX, signature: inflammasomes), 0.796 (TCZ, signature: specific-CD19) and 0.768 (ABT, signature: specific-CD56). Notably, for all biologic agents, the PPVs (positive predictive value) were high (IFX 83.6%, TCZ 92.3%, ABT 94.7%).

Overview of classification of all 209 samples using signature scores

A heatmap using core genes of inflammasomes, specific-CD19 and specific-CD56, revealed all 209 samples analyzed in this study could be distributed into 8 groups, based on the signature score cut-off points determined in ROC analyses (Figure 4). When these groups were compared to actual therapeutic outcomes, the proportions of NON-REM in group 1 and 2 were high for all biologics (80-100%). On the other hand, group 5, 6 and 7 were composed of patients who achieved remission when IFX was administered while group 8 was a group who achieved remission when TCZ or ABT was administered. There were too few patients to draw an observation for group 3 and 4.

Discussion

Most if not all therapeutic effect prediction studies based on gene expression research have been focusing on single rather than multiple biologic agents. Variations of study design of these studies including recruitment criteria of subjects, evaluation of treatment response and platform of assay system represent a huge challenge to combine these findings in translational studies. Therefore, it is important to develop "a unified test platform" which allows a level and concomitant comparison among multiple biologic agents and hence anticipation of the therapeutic outcomes. In this study, we have established a clinically practical system to predict the therapeutic effects of three biologics (IFX, TCZ, and ABT). First, we enrolled RA patients who showed inadequate response to MTX and were administered with one of the three biologics for the first time. Second, we used CDAI to evaluate therapeutic effects so as to minimize bias among the three drugs [12-13]. Third, total RNAs were taken from whole blood with a well-standardized RNA extraction method (PAXgene blood RNA system [14]) and analyzed with a single microarray platform (Agilent microarray).

There is no overlap of gene sets among the three biologics in GSEA, demonstrating that the molecular targets of each biologic are distinct. This finding encouraged us to proceed with this method for comparing other drugs in this platform.

We observed that IFX's NON-REM is typically reflected in their

up-regulated gene expression patterns of inflammasome, which is a multi-protein complex that plays a key role in the production of inflammatory cytokines, such as pro-inflammatory cytokines IL-1β, IL-18 [18]. Inflammasome is associated with pathology of various autoimmune diseases, including RA [19-20].

Takeuchi et al. reported that the amount of IFX required to administer to a patient could be indicated by the baseline TNF protein level in order to achieve an effective response [21]. Moreover, Inflammasome was reported to be activated downstream of TNF signal [22-24]. Therefore, our observation of up-regulated expression of Inflammasome-related genes in NON-REM group of patients, indeed reflects the stimulated TNF signal which could not be attenuated by standard amount of IFX. As a result, a higher dosage of IFX could be a more plausible approach. Differential expression of TNF mRNA between REM and NON-REM was not observed in our analysis as TNF protein is mainly found rather in inflammatory joints than in whole blood. In the future, it would be interesting to delineate the relationship between expression of Inflammasome genes and concentration of TNF in the blood.

For TCZ, we found B cell-related gene set is a promising predictive signature: patients who have a low expression of B cells have poor remission rates. TCZ works as an inhibitor of IL-6 receptor signaling by directly targeting at soluble and membrane-bound IL-6 receptor. IL-6 is an important B cell–stimulating factor and induces antibody synthesis [25], and in RA

pathogenesis, IL-6 induces autoantibody-producing plasma cells [26]. Furthermore, a subset of B cells, especially memory B cell reported to decrease when TCZ was administered to RA patients [27-28]. These findings indicate a close relationship between TCZ response and B cells, as also pointed out by our results. The underlying cause between TCZ's REM and NON-REM could be the ability to regulate the amount of B cells and/or the functional subtypes of B cells (memory B cell) as reflected from the expression difference.

NK cell-related genes comprise significant predictors of ABT's NON-REM: the expression of NK cell-related genes is relatively higher in NON-REM than that of REM. As a component of innate immune system, NK cells are known to regulate activities of dendritic cells, macrophages and T cells [29]. For example, NK cells were demonstrated to negatively regulate self-responsive T cells in various autoimmune disease models [30-32]. A therapy using ABT, which suppresses T cell, to patients expressing high level of NK cell-related genes, which may render activities of T cell being suppressed, could be redundant. It is more likely that there are other contributing factors apart from T cells for this type of patient. However, as pointed out by Shegarfi et al [33], the role of NK cells related to development of RA should worth further delineation.

Core genes found in this study differ from marker genes identified in other studies (IFX: Lequerre et al [2], Tanino et al [3], Julia et al [4], Stuhlmuller et al [5], Cui et al [6], Oswald et al [34], TCZ: Sanayama et al [7]) due to different

evaluation parameters of therapeutic outcome used in each study (DAS28, EULAR criteria etc.), type of samples (whole blood or PMBC) and sample size. The most contributing factor could be the analysis approach. Most biological phenomena especially development of heterogeneous disease like RA, are not a consequence of aberrant individual genes but rather a network of related genes. Therefore we have employed GSEA to capture the biological feature of genes that will provide a robust model to predict the efficacy of biologics. In fact, functional gene set analysis was successful in identification of interferon gene sets as predictor of efficacy of rituximab [8-9].

The performance (AUC of ROC) of predicting therapeutic effect (NON-REM) using the signature score for each drug, i.e., "inflammasomes", "specific-CD19" and "specific-CD56", for IFX, TCZ and ABT, respectively, are 0.637, 0.796, and 0.768, respectively. At the optimal cut-off value from ROC, a notable feature is the high PPV, which was 83.6, 92.3 and 94.7%, for IFX, TCZ and ABT, respectively (Figure 3). In other words, our approach has a unique feature that could indicate accurately which patient would not likely achieve remission. Although it represents an elimination approach rather than selecting a biologic option, it should be equally effective at clinical practical level in the context to increase the probability of a patient to achieve remission. From this approach, we have also discovered a group of patients (Figure 4, group 1), constitute about 20% of patients in this study, who are not likely to achieve remission with either biologic (remission rate is a merely 11.9% (5/42)). Future

studies exploring biologics other than the current 3 biologics or differentiation analysis to predict achievement of low disease activity are essential. However, since ROC analysis was conducted using the same sample group that was used to construct gene signature, overfitting problem might occur. It is essential to validate in independent cohorts in the future.

While this is an observational study conducted under actual clinical setting, it has also inevitably introduced limitations that include bias in clinical background and unmatched number of samples collected for the studied biologics. In fact, IFX was approved long before TCZ and ABT in Japan, which is self-explanatory why IFX outnumbered TCZ and ABT. We believe that the choice of actual clinical settings also led to the bias in clinical background between three biologics, such as ABT group was older than the other two and co-administration with MTX was more likely associated with IFX group. An independent cohort study where clinical background is matched could provide a clearer answer. Another limitation was the significant difference in baseline clinical background between REM and NON-REM. Although we have shown that gene expression signature score remains significant after adjusting the baseline clinical background, again the small number of samples of ABT and TCZ still might not be absolutely persuasive. We are planning to increase the number of samples for validation. Last but not least, as specimen used in this study is RNA extracted from the whole blood, which is composed of various types of blood cells, it is not clear if the gene expression signature is just a

reflection of different amounts of components of blood cells. This remains to be addressed by analyzing immunophenotyping data in the future.

Conclusions

In conclusion, we have succeeded in identifying gene expression signatures for predictions of therapeutic effects of three biologics, IFX, TCZ, and ABT. This represents the first attempt in RA treatment history to address three biologics in a unified test platform. The signatures also meet the latest clinical notions regarding the mode of action of each targeted therapies [22-23, 27-28]. Therefore, the signatures should not be limited to predict therapeutic effects but also at the same time providing bases for future studies on prediction of novel drugs as well as a better classification of RA patients and thus would lead to a better care.

Declarations

Abbreviations

MTX: methotrexate, bDMARDs: biological disease-modifying anti-rheumatic drugs, RA: rheumatoid arthritis, EULAR: The European League Against Rheumatism, TNFi: tumor necrosis factor inhibitor, IFX: infliximab, TCZ: tocilizumab, ABT: abatacept, csDMARDs: conventional synthetic disease-modifying anti-rheumatic drugs, GSEA: gene set enrichment analysis, ACR: The American College of Rheumatology, CDAI: clinical disease activity index, REM: patients with CDAI remission at 6 months of biologic therapy, NON-REM: patients without CDAI remission at 6 months of biologic therapy, DAS28: disease activity score in 28 Joints, CRP: C-reactive protein, ESR: erythrocyte sedimentation rate, LOCF: last observation carried forward, RNA: ribonucleic acid, cRNA: complementary ribonucleic acid, qRT-PCR: quantitative reverse transcription polymerase chain reaction, cDNA: complementary deoxyribonucleic acid, ROC: receiver operating characteristic, AUC: area under the curve, PPV: positive predictive value, NPV: negative predictive value, TJC: tender joint count, SJC: swollen joint count, PtGA: patient's global assessment, PhGA: physician’s global assessment, SDAI: simplified disease activity index, NK cell: natural killer cell, IL: interleukin

Competing interests

SN, HI, YH, CRL, YI, and KM are members of DNA Chip Research Inc. RM is a

chief executive officer of DNA Chip Research Inc. KS has received research grants from Bristol-Myers Squibb, and Eisai Co., Ltd. HK has received grants from AbbVie GK., Astellas Pharma, Chugai Pharmaceutical Co., Ltd., Eisai Co., Ltd., Mitsubishi Tanabe Pharma Co., Pfizer Japan Inc., Santen Pharmaceutical Co., Ltd., and Takeda Pharmaceutical Co., Ltd. Speaking fees from AbbVie GK., Astellas Pharma, Bristol-Myers K.K., Chugai Pharmaceutical Co., Ltd., Eisai Co., Ltd., Janssen Pharmaceutical K.K., Mitsubishi Tanabe Pharma Co., Nippon Kayaku Co., Ltd., Pfizer Japan Inc., Takeda Pharmaceutical Co., Ltd., and UCB Pharma. Consultant fees from AbbVie GK., Eli Lilly Japan K.K., Novartis Pharma K.K., Sanofi Pharma, and Nippon Kayaku Co., Ltd. KA has received grants from Astellas Pharma, Chugai Pharmaceutical Co., Ltd., Mitsubishi Tanabe Pharma Co., and Pfizer Japan Inc. Speaking fees from AbbVie GK., Astellas Pharma, Bristol-Myers K.K., Chugai Pharmaceutical Co., Ltd., Mitsubishi Tanabe Pharma Co., and Pfizer Japan Inc. TT has received grants from AbbVie GK., Asahi Kasei Pharma Corp., Astellas Pharma, Bristol-Myers K.K., Chugai Pharmaceutical Co., Ltd., Daiichi Sankyo Co., Ltd., Eisai Co., Ltd., Mitsubishi Tanabe Pharma Co., Pfizer Japan Inc., Santen Pharmaceutical Co., Ltd., SymBio Pharmaceuticals Ltd., Takeda Pharmaceutical Co., Ltd., Taisho Toyama Pharmaceutical Co., Ltd., and Teijin Pharma Ltd. Speaking fees from AbbVie GK., Astellas Pharma, Bristol-Myers K.K., Celltrion, Inc., Chugai Pharmaceutical Co., Ltd., Daiichi Sankyo Co., Ltd., Eisai Co., Ltd., Janssen Pharmaceutical K.K., Mitsubishi Tanabe Pharma Co., Nippon Kayaku Co., Ltd.,

Pfizer Japan Inc., and Takeda Pharmaceutical Co., Ltd. Consultant fees from AbbVie GK., Asahi Kasei Medical K.K., Astra Zeneca K.K., Bristol–Myers K.K., Daiichi Sankyo Co., Ltd., Eli Lilly Japan K.K., Mitsubishi Tanabe Pharma Co., Nippon Kayaku Co., Ltd., and Novartis Pharma K.K. The authors have no non-financial conflicts of interest.

Authors' contributions

SN analyzed the microarray and clinical data, performed interpretation of data, and drafted the manuscript. KS supplied patient samples, collection of clinical data, and critically revised the manuscript. HI analyzed the microarray data, performed the statistical analysis and helped to draft the manuscript. YH analyzed the clinical data performed the statistical analysis and helped to draft the manuscript. CRL analyzed the microarray and clinical data, and critically revised the manuscript. YI performed the microarray experiments and helped to draft the manuscript. HK supplied patient samples, collection of clinical data, and revised the manuscript. KA supplied patient samples, collection of clinical data, and revised the manuscript. KM conceived of the study, performed interpretation of data, and critically revised the manuscript. RM participated in its design and coordination, and revised manuscript. TT conceived of the study, participated in its design and coordination, and critically revised manuscript. All authors read and approved to the final version of the manuscript.

Acknowledgments

We would like to thank the patients and staffs who participated in this study. We also thank Ms. Sumiko Kaihara for English corrections of the original draft. This work was supported by the subsidies from Japan's New Energy and Industrial Technology Development Organization and supported in part by funding from DNA Chip Research Inc.

References

1. Smolen JS, Landewe R, Breedveld FC, Buch M, Burmester G, Dougados M et al. EULAR recommendations for the management of rheumatoid arthritis with synthetic and biological disease-modifying antirheumatic drugs: 2013 update. Ann Rheum Dis. 2014;73:492-509.
2. Lequerre T, Gauthier-Jauneau AC, Bansard C, Derambure C, Hiron M, Vittecoq O et al. Gene profiling in white blood cells predicts infliximab responsiveness in rheumatoid arthritis. Arthritis Res Ther. 2006;8:R105.
3. Tanino M, Matoba R, Nakamura S, Kameda H, Amano K, Okayama T et al. Prediction of efficacy of anti-TNF biologic agent, infliximab, for rheumatoid arthritis patients using a comprehensive transcriptome analysis of white blood cells. Biochem Biophys Res Commun. 2009;387:261-265.
4. Julia A, Erra A, Palacio C, Tomas C, Sans X, Barcelo P et al. An eight-gene blood expression profile predicts the response to infliximab in rheumatoid arthritis. PLoS One. 2009;4:e7556.
5. Stuhlmuller B, Haupl T, Hernandez MM, Grutzkau A, Kuban RJ, Tandon N et al. CD11c as a transcriptional biomarker to predict response to anti-TNF monotherapy with adalimumab in patients with rheumatoid arthritis. Clin Pharmacol Ther. 2010;87:311-321.
6. Cui J, Stahl EA, Saevarsdottir S, Miceli C, Diogo D, Trynka G et al. Genome-wide association study and gene expression analysis identifies CD84 as a predictor of response to etanercept therapy in rheumatoid arthritis. PLoS Genet. 2013;9:e1003394.
7. Sanayama Y, Ikeda K, Saito Y, Kagami S, Yamagata M, Furuta S et al. Prediction of therapeutic responses to tocilizumab in patients with rheumatoid arthritis: biomarkers identified by analysis of gene expression in peripheral blood mononuclear cells using genome-wide DNA microarray. Arthritis Rheumatol. 2014;66:1421-1431.
8. Thurlings RM, Boumans M, Tekstra J, van Roon JA, Vos K, van Westing DM et al. Relationship between the type I interferon signature and the response to rituximab in rheumatoid arthritis patients. Arthritis Rheum. 2010;62:3607-3614.
9. Raterman HG, Vosslamber S, de Ridder S, Nurmohamed MT, Lems WF, Boers M et al. The interferon type I signature towards prediction of non-response to rituximab in rheumatoid arthritis patients. Arthritis Res Ther. 2012;14:R95.
10. Burska AN, Roget K, Blits M, Soto Gomez L, van de Loo F, Hazelwood LD et al. Gene expression analysis in RA: towards personalized medicine. Pharmacogenomics J. 2014;14:93-106.
11. Subramanian A, Tamayo P, Mootha VK, Mukherjee S, Ebert BL, Gillette MA et al. Gene set enrichment analysis: a knowledge-based approach for

interpreting genome-wide expression profiles. Proc Natl Acad Sci U S A. 2005;102:15545-15550.
12. Kawashiri SY, Kawakami A, Iwamoto N, Fujikawa K, Aramaki T, Tamai M et al. Disease activity score 28 may overestimate the remission induction of rheumatoid arthritis patients treated with tocilizumab: comparison with the remission by the clinical disease activity index. Mod Rheumatol. 2011;21:365-369.
13. Smolen JS, Aletaha D. Interleukin-6 receptor inhibition with tocilizumab and attainment of disease remission in rheumatoid arthritis: the role of acute-phase reactants. Arthritis Rheum. 2011;63:43-52.
14. Rainen L, Oelmueller U, Jurgensen S, Wyrich R, Ballas C, Schram J et al. Stabilization of mRNA expression in whole blood samples. Clin Chem. 2002;48:1883-1890.
15. Watkins NA, Gusnanto A, de Bono B, De S, Miranda-Saavedra D, Hardie DL et al. A HaemAtlas: characterizing gene expression in differentiated human blood cells. Blood. 2009;113:e1-9.
16. Allantaz F, Cheng DT, Bergauer T, Ravindran P, Rossier MF, Ebeling M et al. Expression profiling of human immune cell subsets identifies miRNA-mRNA regulatory relationships correlated with cell type specific expression. PLoS One. 2012;7:e29979.
17. Firth D. Bias reduction of maximum likelihood estimates. Biometrika. 1993;80:27-38.
18. Schroder K, Tschopp J. The inflammasomes. Cell. 2010;140:821-832.
19. Rosengren S, Hoffman HM, Bugbee W, Boyle DL. Expression and regulation of cryopyrin and related proteins in rheumatoid arthritis synovium. Ann Rheum Dis. 2005;64:708-714.
20. Shaw PJ, McDermott MF, Kanneganti TD. Inflammasomes and autoimmunity. Trends Mol Med. 2011;17:57-64.
21. Takeuchi T, Miyasaka N, Tatsuki Y, Yano T, Yoshinari T, Abe T et al. Baseline tumour necrosis factor alpha levels predict the necessity for dose escalation of infliximab therapy in patients with rheumatoid arthritis. Ann Rheum Dis. 2011;70:1208-1215.
22. Alvarez S, Munoz-Fernandez MA. TNF-Alpha may mediate inflammasome activation in the absence of bacterial infection in more than one way. PLoS One. 2013;8:e71477.
23. Brennan FM, Chantry D, Jackson A, Maini R, Feldmann M. Inhibitory effect of TNF alpha antibodies on synovial cell interleukin-1 production in rheumatoid arthritis. Lancet. 1989;2:244-247.
24. Feldmann M, Brennan FM, Maini RN. Role of cytokines in rheumatoid arthritis. Annu Rev Immunol. 1996;14:397-440.
25. Suematsu S, Matsuda T, Aozasa K, Akira S, Nakano N, Ohno S et al. IgG1 plasmacytosis in interleukin 6 transgenic mice. Proc Natl Acad Sci U S A. 1989;86:7547-7551.

26. Ohsugi Y, Kishimoto T. The recombinant humanized anti-IL-6 receptor antibody tocilizumab, an innovative drug for the treatment of rheumatoid arthritis. Expert Opin Biol Ther. 2008;8:669-681.

27. Roll P, Muhammad K, Schumann M, Kleinert S, Einsele H, Dorner T et al. In vivo effects of the anti-interleukin-6 receptor inhibitor tocilizumab on the B cell compartment. Arthritis Rheum. 2011;63:1255-1264.

28. Kikuchi J, Hashizume M, Kaneko Y, Yoshimoto K, Nishina N, Takeuchi T. Peripheral blood CD4(+)CD25(+)CD127(low) regulatory T cells are significantly increased by tocilizumab treatment in patients with rheumatoid arthritis: increase in regulatory T cells correlates with clinical response. Arthritis Res Ther. 2015;17:10.

29. Malhotra A, Shanker A. NK cells: immune cross-talk and therapeutic implications. Immunotherapy. 2011;3:1143-1166.

30. Matsumoto Y, Kohyama K, Aikawa Y, Shin T, Kawazoe Y, Suzuki Y et al. Role of natural killer cells and TCR gamma delta T cells in acute autoimmune encephalomyelitis. Eur J Immunol. 1998;28:1681-1688.

31. Fort MM, Leach MW, Rennick DM. A role for NK cells as regulators of CD4+ T cells in a transfer model of colitis. J Immunol. 1998;161:3256-3261.

32. Trivedi PP, Roberts PC, Wolf NA, Swanborg RH. NK cells inhibit T cell proliferation via p21-mediated cell cycle arrest. J Immunol. 2005;174:4590-4597.

33. Shegarfi H, Naddafi F, Mirshafiey A. Natural killer cells and their role in rheumatoid arthritis: friend or foe? ScientificWorldJournal. 2012;2012:491974.

34. Oswald M, Curran ME, Lamberth SL, Townsend RM, Hamilton JD, Chernoff DN et al. Modular analysis of peripheral blood gene expression in rheumatoid arthritis captures reproducible gene expression changes in tumor necrosis factor responders. Arthritis Rheumatol. 2015;67:344-351.

Tables

Table 1 Baseline demographics and characteristics of patients.

		ALL	IFX	TCZ	ABT	p-Value[1]
**Number**	**n**	209	140	38	31	-
**Female**	**n (%)**	172 (82.3%)	113 (80.7%)	34 (89.5%)	25 (80.6%)	0.46626
**Age, yrs**	**median (IQR)**	59.0 (47.0, 66.0)	57.5 (46.0, 64.3)	56.0 (44.8, 64.0)	67.0 (62.0, 74.0)	0.00003
**Disease duration, yrs**	**median (IQR)**	3.3 (1.1, 10.5)	3.3 (1.1, 10.3)	4.2 (1.5, 9.4)	2.4 (0.5, 14.5)	0.86013
**Concomitant drug use**						
**Steroid use**	**n (%)**	80 (38.3%)	56 (40.0%)	14 (36.8%)	10 (32.3%)	0.75247
**Steroid dose, mg/day**	**median (IQR)**	0 (0, 5.0)	0 (0, 5.0)	0 (0, 3.0)	0 (0, 2.5)	0.34861
**MTX dose, mg/week**	**median (IQR)**	8.0 (8.0, 8.0)	8.0 (8.0, 10.0)	8.0 (6.0, 8.0)	8.0 (6.0, 8.0)	0.00271
**csDMARDs use (except MTX)[2]**	**n (%)**	29 (13.9%)	20 (14.3%)	5 (13.2%)	4 (12.9%)	1.00000
**Serological markers**						
**RF positivity**	**n (%)**	154 (74.8%)[3]	103 (74.1%)[4]	28 (73.7%)	23 (79.3%)[5]	0.88544
**RF titer**	**median (IQR)**	55 (15, 115)[3]	53 (14, 115)[4]	54 (17, 115)	77 (22, 106)[5]	0.94307
**ACPA positivity**	**n (%)**	85 (85.9%)[6]	51 (85.0%)[7]	13 (100%)[8]	21 (80.8%)[9]	0.31205
**ACPA antibody titer**	**median (IQR)**	85.6 (12.8, 100)[6]	100 (13.3, 100)[7]	83 (42.9, 100)[8]	62.1 (9.8, 100)[9]	0.89773
**TJC28**	**median (IQR)**	6.0 (2.0, 8.0)	6.0 (2.0, 8.3)	6.0 (3.0, 8.8)	6.0 (3.0, 8.0)	0.68798
**SJC28**	**median (IQR)**	6.0 (3.0, 11.0)	7.0 (3.8, 11.0)	6.0 (4.0, 8.0)	5.0 (3.0, 8.0)	0.46376
**PtGA, mm**	**median (IQR)**	53 (28, 72)	52 (27, 72)	52 (31, 67)	63 (41, 73)	0.34811
**PhGA, mm**	**median (IQR)**	43 (30, 60)	43 (29, 63)	43 (33, 60)	45 (31, 56)	0.91700
**CRP, mg/dl**	**median (IQR)**	0.9 (0.4, 2.4)	1.0 (0.4, 2.6)	0.6 (0.2, 2.0)	0.8 (0.4, 2.0)	0.19092
**ESR, mm/h**	**median (IQR)**	42 (25, 69)	45 (28, 69)	37 (22, 60)	37 (23, 74)	0.27517
**DAS28-ESR**	**median (IQR)**	5.3 (4.4, 6.1)	5.3 (4.4, 6.2)	5.1 (4.5, 5.8)	5.5 (4.7, 6.1)	0.65899
**SDAI score**	**median (IQR)**	22.5 (16.2, 31.3)	23.2 (15.6, 32.2)	22.1 (16.8, 28.5)	23.3 (16.8, 31.5)	0.96973
**CDAI score**	**median (IQR)**	21.7 (14.9, 28.9)	21.9 (14.6, 29.6)	20.6 (16.4, 26.0)	22.8 (15.1, 28.5)	0.99430

[1] Kruskal–Wallis test was used for numerical variables to evaluate the differences between the three drug groups. For categorical variables, Fisher's exact test was used. p-Value < 0.05 was considered statistically significant.

[2] Including salazosulfapyridine, bucillamin, tacrolimus, D-penicillamine, actarit, azathioprine.

[3] Available for 206 out of 209. [4] Available for 139 out of 140. [5] Available for 29 out of 31. [6] Available for 99 out of 209. [7] Available for 60 out of 140. [8] Available for 13 out of 38. [9] Available for 26 out of 31.

IQR, interquartile range; MTX, methotrexate; csDMARDs, conventional synthetic disease-modifying antirheumatic drugs; RF, rheumatoid factor; ACPA, anti-cyclic citrullinated peptide antibodies; TJC, tender joint count; SJC, swollen joint count; PtGA, patient global assessment; PhGA, physician global assessment; CRP, C-reactive protein; ESR, erythrocyte sedimentation rate; DAS, disease activity score; SDAI, simplified disease activity index; CDAI, clinical disease activity index.

Table 2 GSEA results.

Gene sets category	Drug	Direction of regulation	Gene set name	SIZE[1]	NES[2]	NOM p-val[3]	FDR q-val[4]
Reactome gene sets	IFX	REM > NON-REM	-	-	-	-	-
		NON-REM > REM	Inflammasomes (M1072)	16	1.95	<0.00001	0.07489
	TCZ	REM > NON-REM	-	-	-	-	-
		NON-REM > REM	-	-	-	-	-
	ABT	REM > NON-REM	-	-	-	-	-
		NON-REM > REM	Elongation arrest and recovery (M810)	27	1.91	0.00180	0.08880
			Regulation of apoptosis (M733)	54	1.96	0.00192	0.09083
			Formation of RNA pol II elongation complex (M805)	38	1.85	<0.00001	0.09138
Blood cell gene sets	IFX	REM > NON-REM	-	-	-	-	-
		NON-REM > REM	-	-	-	-	-
	TCZ	REM > NON-REM	Specific-CD19 (Watkins et al. 2009)	140	-1.70	0.00602	0.02646
			B cells-induced (Allantaz et al. 2012)	57	-1.56	0.01603	0.08882
		NON-REM > REM	-	-	-	-	-
	ABT	REM > NON-REM	-	-	-	-	-
		NON-REM > REM	Specific-CD56 (Watkins et al. 2009)	51	1.60	0.02390	0.02615
			NK cells-induced (Allantaz et al. 2012)	78	1.63	0.00403	0.02861

[1] Number of genes found in the gene set from expression dataset.
[2] Normalized enrichment score; the enrichment score for the gene set after it has been normalized across analyzed gene sets.
[3] Nominal p-value; that is the statistical significance of the enrichment score. Nominal p-value is not adjusted for gene set size or multiple hypothesis testing.
[4] False discovery rate; that is the estimated probability that the normalized enrichment score represents a false positive finding.
A NOM p-val < 0.05 and a FDR q-val < 0.1 was considered statistically significant.
REM, patients with CDAI remission* at 6 months of biologic therapy; NON-REM, patients without CDAI remission* at 6 months of biologic therapy.
* CDAI remission is defined as CDAI <= 2.8.

Table 3 Logistic regression analyses using signature scores to predict CDAI non-remission at 6 months of biologic therapy.

Drug	Gene set	Univariate analysis			Multivariate analysis[1]		
		ORs	95% CI	p-Value	ORs	95% CI	p-Value
IFX							
	Inflammasomes	1.81	(1.23, 2.78)	0.00382	1.72	(1.14, 2.71)	0.00873
TCZ							
	Specific-CD19 (Watkins 2009)	0.24	(0.05, 0.72)	0.02677	0.16	(0.02, 0.71)	0.01327
ABT							
	Elongation arrest and recovery	5.73	(1.47, 49.11)	0.04488	6.85	(1.09, 4426.71)	0.03309
	Specific-CD56 (Watkins 2009)	3.25	(1.18, 12.43)	0.04179	6.46	(1.60, 88.39)	0.00388

[1] Multivariate analysis adjusted for significant (p<0.1) variables as in Additional file 3 (IFX: female, concomitant csDMARDs use (except MTX), ESR, CDAI; TCZ: disease duration, CDAI; ABT: disease duration, concomitant steroid use).
OR, odds ratio; CI, confidence interval.

Figure legends

Figure 1 Disease activity based on CDAI at baseline and 6 months of biologic therapy.

LOCF, last observation carried forward.

Figure 2 Comparisons of signature scores between REM and NON-REM in IFX (A), TCZ (B, C), and ABT (D-H).

Distribution of the values, mean, upper and lower limit of 95% confidence interval (CI) for the mean are shown. p-Values were determined using Student's t-test. p-Value < 0.05 was considered statistically significant. REM, patients with CDAI remission* at 6 months of biologic therapy; NON-REM, patients without CDAI remission* at 6 months of biologic therapy.

* CDAI remission is defined as CDAI <= 2.8.

Figure 3 ROC analyses for the prediction of CDAI non-remission at 6 months of biologic therapy (IFX (A), TCZ (B), and ABT (C)).

AUC, area under the curve; CI, confidence interval; PPV, positive* predictive value; NPV, negative predictive value. * "Positive" means CDAI non-remission at 6 months of biologic therapy.

Figure 4 An overview of classification of all 209 samples using three signature scores.

All 209 samples could be classified in eight groups, based on the binary variables derived from the signature scores of the three gene sets, i.e. inflammasomes-, specific-CD19-, and specific-CD56-related. The threshold for binary call was determined using ROC analysis.

(Upper panel) Heatmap of 209 samples based on expression patterns of the three core gene sets. Heatmap drawing was performed based on relative expression levels (Z-scores) of core genes using TIGR MultiExperiment Viewer Software (http://www.tm4.org/). (Middle panel) Prediction results using signature scores. Patients were predicted using signature score and grouped as remission “REM” and non-remission “NON-REM” as indicated in green and red, respectively. (Bottom panel) Actual remission status based on CDAI at sixth month. In ″Individual outcome″, green- or red-filled circle represent actual individual case achieving or not achieving remission, respectively. In ″Rate of NON-REM″, the actual number of cases and the non-remission rates are indicated for all eight groups.

Additional files

Additional file 1 Blood cell type-specific expressed gene sets. (XLSX 66kb)

Additional file 2 Procedure of signature score calculation. (PDF 203kb)

Additional file 3 Comparisons of baseline characteristics between REM and NON-REM. (XLSX 18kb)

Additional file 4 Illustration of the distribution of patients in this observational study. (PDF 224kb)

Additional file 5 Enrichment-plots of gene sets extracted from GSEA. (PDF 406kb)

Additional file 6 Ranked gene list by GSEA (IFX analysis). (XLSX 903kb)

Additional file 7 Ranked gene list by GSEA (TCZ analysis). (XLSX 985kb)

Additional file 8 Ranked gene list by GSEA (ABT analysis). (XLSX 1152kb)

Additional file 9 qRT-PCR results of 5 core genes of each signature identified from GSEA. (PDF 318kb)

Additional file 10 Correlation matrixes of signature scores of core genes for TCZ (A) and ABT (B). (PDF 298kb)

 Click here to download Figure Figure 1.tiff

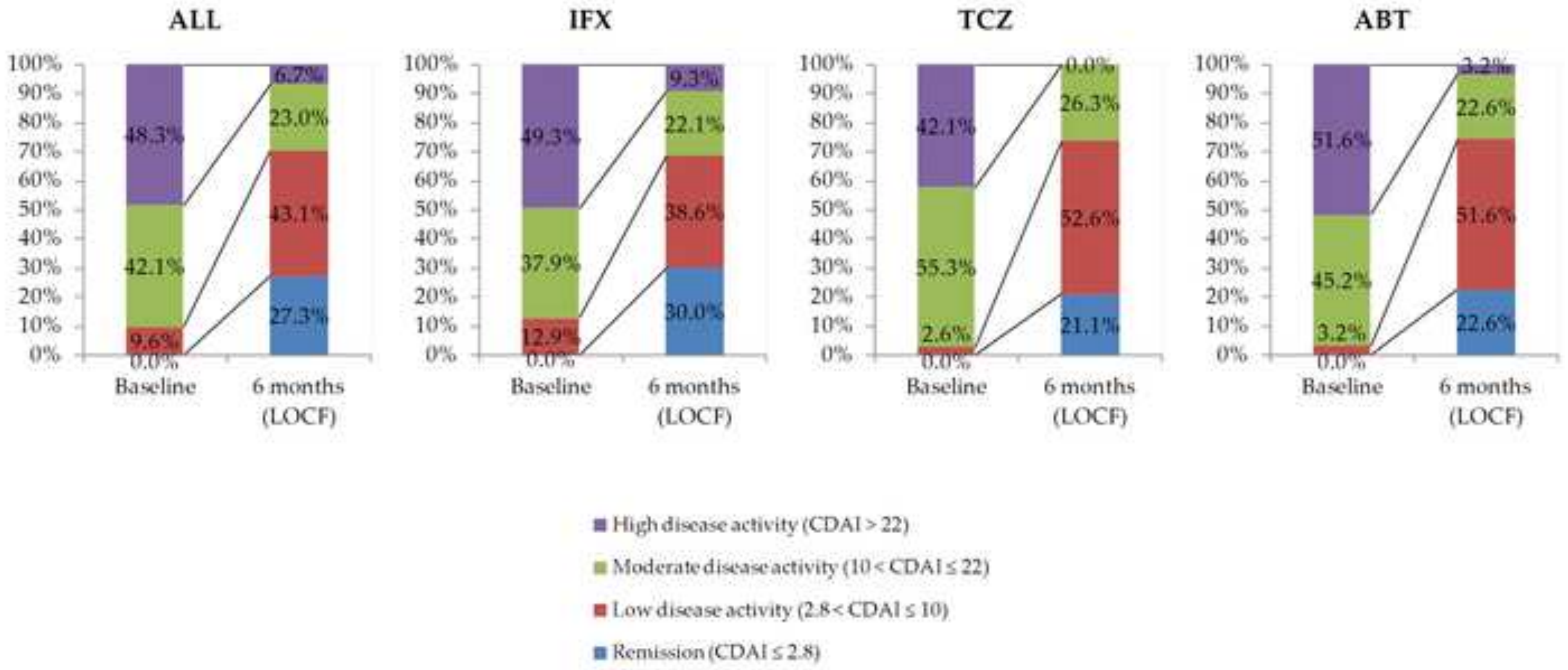

Figure 2

Click here to download Figure Figure 2.tiff

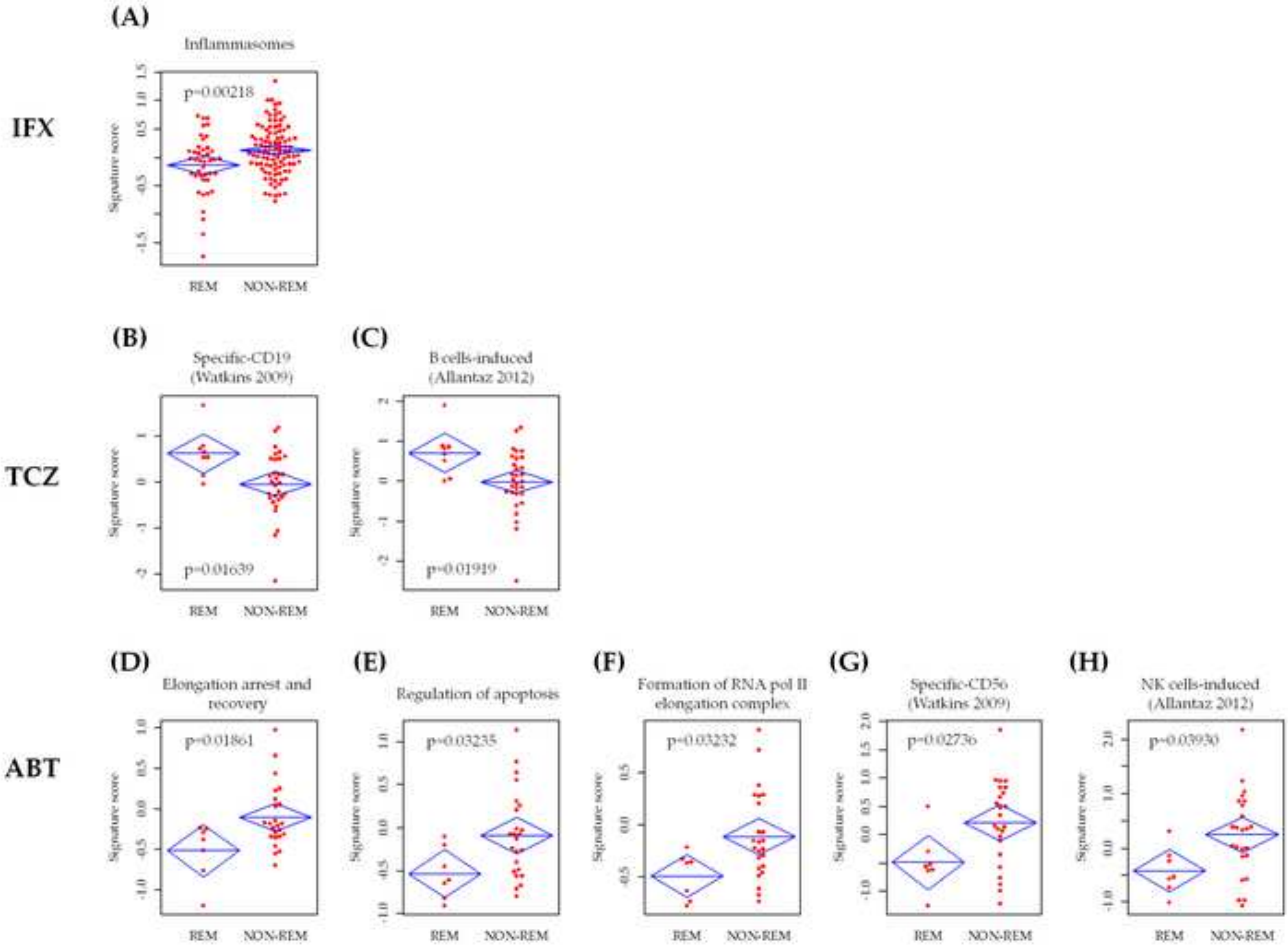

Figure 3

Click here to download Figure Figure 3.tiff

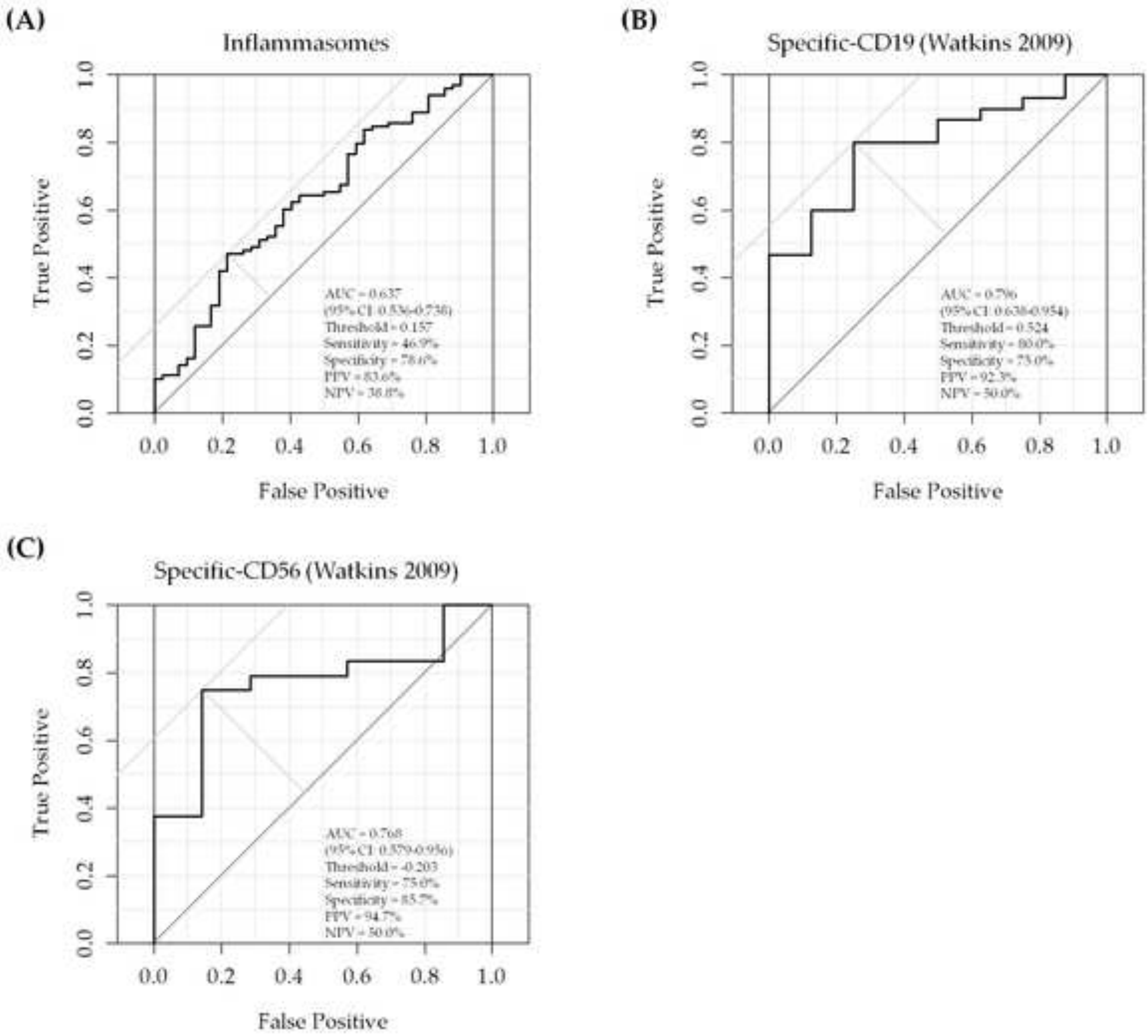

Figure 4

Click here to download Figure Figure 4.tiff

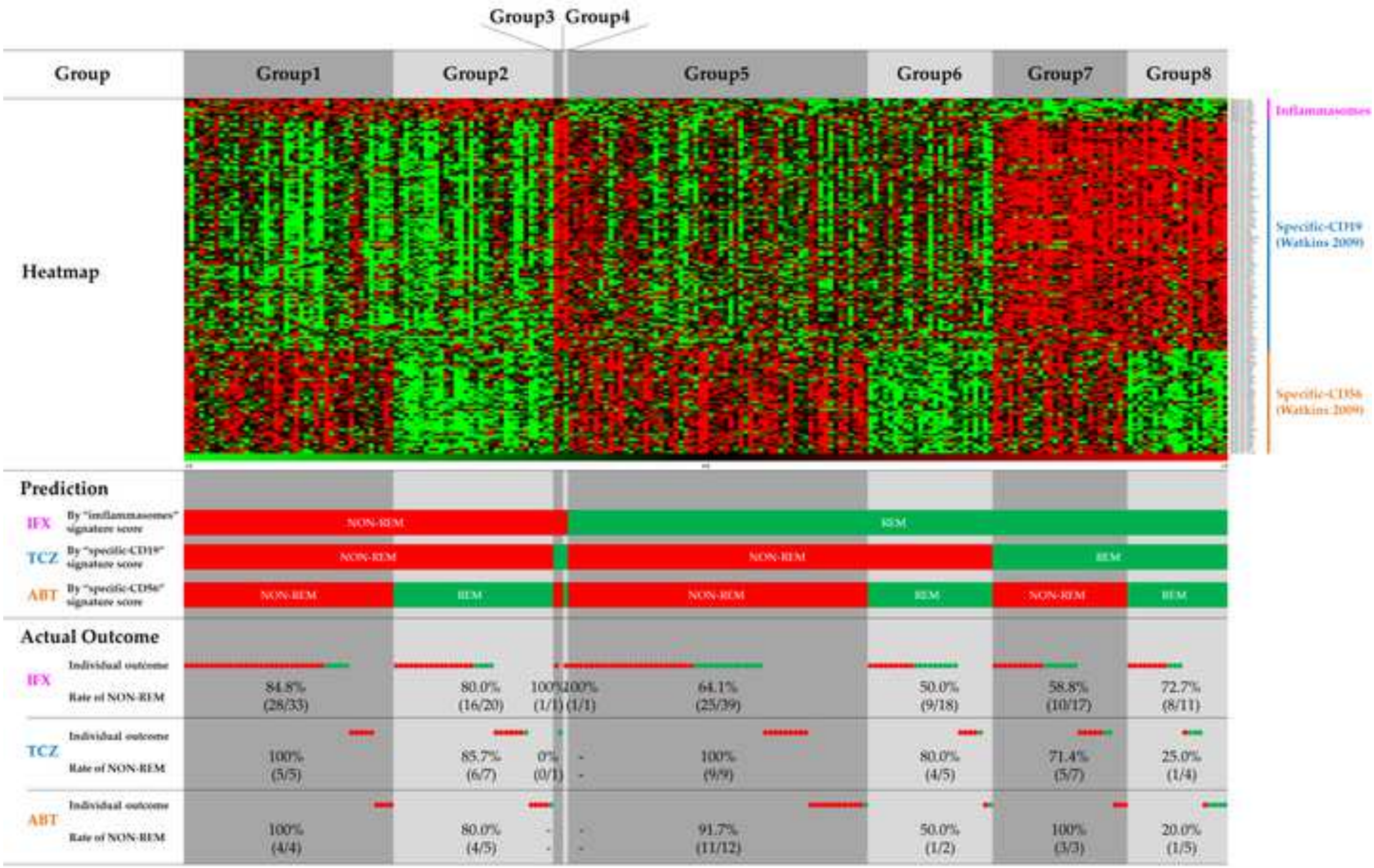

Additional file 1

Click here to access/download

Supplementary Material

Additional file 1.xlsx

Additional file 2

Click here to access/download
Supplementary Material
Additional file 2.pdf

Additional file 3

Click here to access/download
Supplementary Material
Additional file 3.xlsx

Additional file 4

Click here to access/download
Supplementary Material
Additional file 4.pdf

Additional file 5

Click here to access/download
Supplementary Material
Additional file 5.pdf

Additional file 6

Click here to access/download
Supplementary Material
Additional file 6.xlsx

Additional file 7

Click here to access/download
Supplementary Material
Additional file 7.xlsx

Additional file 8

Click here to access/download
Supplementary Material
Additional file 8.xlsx

Additional file 9

Click here to access/download
Supplementary Material
Additional file 9.pdf

Additional file 10

Click here to access/download
Supplementary Material
Additional file 10.pdf

Personal Cover_revised Click here to download Personal Cover Cover letter_revised.docx

Professor Kazuhiko Yamamoto, University of Tokyo, Japan

Section editor of genetics and epigenetics, *Arthritis Research & Therapy*

June 3, 2016

Ref.: ARRT-D-16-00110

Identification of baseline gene expression signatures predicting therapeutic responses to three biologic agents in rheumatoid arthritis: a retrospective observational study

Dear Professor Kazuhiko Yamamoto;

Thank you for your email of May 12, 2016, regarding our manuscript, "Identification of baseline gene expression signatures predicting therapeutic responses to three biologic agents in rheumatoid arthritis: a retrospective observational study", and the valuable additional comments of the reviewer #1. I attach here our revised manuscript (the yellow highlighted characters are places of correction), as well as a point-by-point response to the reviewer's comments. We have extensively revised our manuscript according to the reviewers' suggestions.

We feel that now the revised manuscript has addressed the points raised by each reviewer, and is significantly improved compared to initial submission. We truly hope that it is now suitable to be considered for publication in *Arthritis Research & Therapy*.

Thank you in advance for your time and kind consideration of this paper.

We look forward to hearing from you regarding our submission. We would be glad to respond to any further questions and comments that you may have.

Sincerely yours,

Seiji Nakamura

DNA Chip Research Inc., 1-15-1 Kaigan, Suzuebaydium 5F, Minato-ku, Tokyo 105-0022, Japan

E-mail : nakamura@dna-chip.co.jp

Tel : + 81-3-5777-1700.

Fax : + 81-3-1689-1687.

RESPONSE TO REVIEWER #1:

Point 1: Although the new text addresses the point that I raised, the text is difficult to understand. Specific details are: the reference to RA as a malignancy, the meaning of "standards used to predict efficacy", and of "total number of 2 cohorts analyzed..." Other sentences that are difficult to understand are those making reference to Oswald et al., and mentioning different approaches for analysis.

Response: By referring to Oswald et al., (an important paper recommended by reviewer #2) at the end of the paragraph, we have incorporated expressions that require explanations. We have therefore moved the reference to the beginning of the paragraph so that it is presented together with other references of previous studies addressing similar topic. Furthermore, we have also rephrased the expressions above.

Before revision

Core genes found in this study differ from other studies (TNFi: Lequerre et al [2], Tanino et al [3], Julia et al [4], Stuhlmuller et al [5], Cui et al [6], TCZ: Sanayama et al [7]) mainly due to the standards used to predict efficacy, type of samples (whole blood or peripheral mononuclear cells) and number of samples used. The most contributing factor could be the analysis approach. Most biological phenomena especially development of a malignancy as heterogeneous as RA, are not a consequence of aberrant individual genes but rather a network of related genes. Therefore we have employed GSEA to capture the feature of genes that will provide a robust model to predict the efficacy of biologics. In fact, while reproducibility was poor using individual genes approach, functional gene set analysis was successful in identification of interferon gene sets as predictor of efficacy of rituximab [8-9]. On the other hand, modular expression approach by Oswald et al was not able to capture gene modules that predict therapeutic effects of different drugs of TNFi [33]. Differences in recruitment criteria of samples (whether they were first biologic and MTX-failed) and also evaluation standards of treatment outcome render direct comparison with current study difficult. In addition, total number of 2 cohorts analyzed for IFX (n=43), the only biologic shared with this study, is arguably smaller than the number of current study (n=140).

After revision (p. 19, line 20 - p. 20, line 9)

Core genes found in this study differ from marker genes identified in other studies (IFX: Lequerre et al [2], Tanino et al [3], Julia et al [4], Stuhlmuller et al [5], Cui et al [6], Oswald

et al [34], TCZ: Sanayama et al [7]) due to different evaluation parameters of therapeutic outcome used in each study (DAS28, EULAR criteria etc.), type of samples (whole blood or PMBC) and sample size. The most contributing factor could be the analysis approach. Most biological phenomena especially development of heterogeneous disease like RA, are not a consequence of aberrant individual genes but rather a network of related genes. Therefore we have employed GSEA to capture the biological feature of genes that will provide a robust model to predict the efficacy of biologics. In fact, functional gene set analysis was successful in identification of interferon gene sets as predictor of efficacy of rituximab [8-9].

Point 2: I fear my comment on this point was not clear. What I intended was to request information on the criteria used to treat some of the patients with Tocilizumab and Abatacept. This needs explanation because none of these two drugs was a first line biologic at the time the patients were treated.

Response: Administration of each biologic is based on JCR guidelines (http://www.ryumachi-jp.com/guideline.html). We have revised the sentences to deliver the points accordingly.

Before revision

Eligible patients were those who met the 1987 revised criteria of the American College of Rheumatology (ACR) for the classification of RA or the 2010 ACR/EULAR classification criteria and responded inadequately (clinical disease activity index: CDAI > 2.8) to MTX (> 6mg/week). RA patients who were commenced with any one of IFX, TCZ, and ABT as their first biologic between May 2007 and November 2011 at Keio University Hospital and Saitama Medical University Saitama Medical Center were enrolled. IFX, TCZ, and ABT were approved as first line biologics in Japan in 2003, 2008 and 2010, respectively and selection was based on Japanese clinical guidelines in parallel with physician's judgement.

After revision (p. 7, line 3 - 11)

The diagnosis of RA was based on the 1987 revised criteria of the American College of Rheumatology (ACR) for the classification of RA or the 2010 ACR/EULAR classification criteria. RA patients who responded inadequately to MTX (>= 6mg/week) and were commenced with any one of IFX, TCZ (2008-), and ABT (2010-) as their first biologic between May 2007 and November 2011 at Keio University Hospital and Saitama Medical University Saitama Medical Center were enrolled. Biologics were administered according to the guidelines set by Japan College of Rheumatology (JCR)

(http://www.ryumachi-jp.com/guideline.html).

Point 13: Quantile normalization does not protect against batch effects. Similarly, validation of top findings is not sufficient warranty. I think a satisfactory evidence would be negative results in any method applied to check for batch effects like cluster analysis or PCA. If batch effects are present, they should be corrected. The most common approach is to use Combat.

Response: Please find results of Hierarchical clustering of samples after Quantile normalization.

(A)

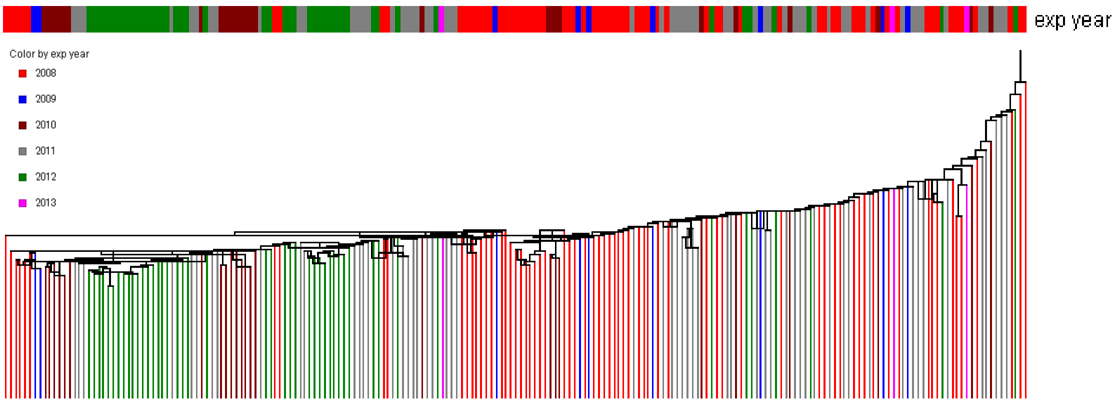

(B)

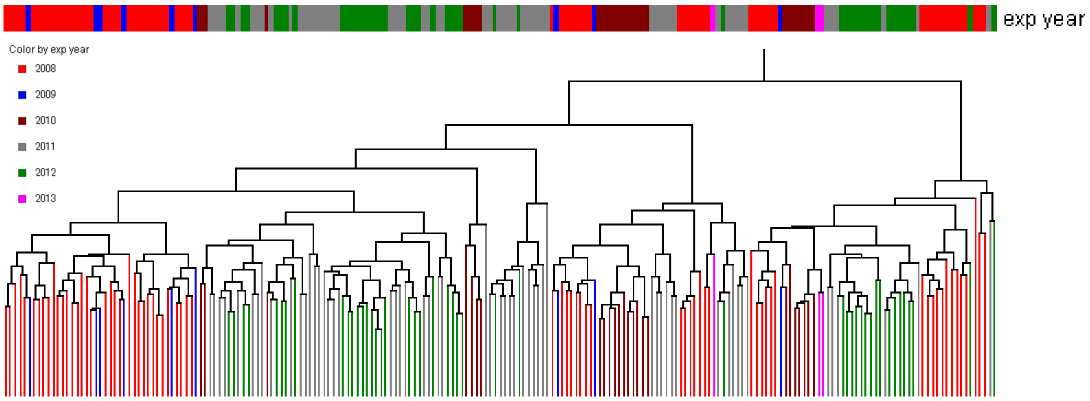

Microarray experiment of current study was performed accordingly as blood samples of study subjects were recruited from year 2008 to 2013. In this figure, we have therefore color-labeled the samples according to the year when data were obtained. Cluster analysis results are often dependent on the algorithms used and hence we have applied 2 types of algorithms, i.e. A Similarity Measure: Euclidean, Linkage Rule: Centroid, B

Similarity Measure: Euclidean, Linkage Rule: Complete, to find if results are consistent.

No particular cluster was observed according to the timing when experiments were conducted for both algorithms and this leads us to conclude that batch effects are negligible. Furthermore, as shown in the previous revision, the signatures obtained from microarray data are reproducible using another methodology, qPCR, and this should solidify the features of signatures we obtained in this study.

Point 17: The paragraph on discussion about NK cells and CD86 in the response to Abatacept is very speculative and I think it should be deleted.

Response: Thank you for your advice. We have replaced the discussion for NK cells as below.

Before revision

NK cell-related genes comprise significant predictors of ABT's NON-REM: the expression of NK cell-related genes is relatively higher in NON-REM than that of REM. NK cells have a close relationship with CD86, the target of ABT. IFNγ from NK cells or NK cells themselves are responsible for the maturing of dendritic cells which express CD86 [29-31]. NK cells were also reported to express CD86 [32]. The up-regulated expression pattern of NK cells gene sets and IFNγ gene (Additional file 8) could be related to activation of NK cells or increase in the number of NK cells, which trigger excessive production of CD86. This may lead to insufficient amount of ABT that has been administered to patients with up-regulated expression pattern of NK cells, to suppress disease activity. The roles of NK cells in the physiological pathology of RA are certainly a field awaiting for more research.

After revision (p. 19, line 8 - 19)

NK cell-related genes comprise significant predictors of ABT's NON-REM: the expression of NK cell-related genes is relatively higher in NON-REM than that of REM. As a component of innate immune system, NK cells are known to regulate activities of dendritic cells, macrophages and T cells [29]. For example, NK cells were demonstrated to negatively regulate self-responsive T cells in various autoimmune disease models [30-32]. A therapy using ABT, which suppresses T cell, to patients expressing high level of NK cell-related genes, which may render activities of T cell being suppressed, could be redundant. It is more likely that there are other contributing factors apart from T cells for this type of patient. However, as pointed out by Shegarfi et al [33], the role of NK cells related to development of RA should worth further delineation.
